# Caffeine-Cyclodextrin Complexes as Solids: Synthesis, Biological and Physicochemical Characterization †

**DOI:** 10.3390/ijms22084191

**Published:** 2021-04-18

**Authors:** Sebastian Szmeja, Tomasz Gubica, Andrzej Ostrowski, Aldona Zalewska, Łukasz Szeleszczuk, Katarzyna Zawada, Monika Zielińska-Pisklak, Krzysztof Skowronek, Małgorzata Wiweger

**Affiliations:** 1 Department of Physical Chemistry, Physical Pharmacy and Bioanalysis, Faculty of Pharmacy, Medical University of Warsaw, Banacha 1, 02-097 Warsaw, Poland; s.szmeja@gmail.com (S.S.); lszeleszczuk@wum.edu.pl (Ł.S.); kzawada@wum.edu.pl (K.Z.); 2 Faculty of Chemistry, Warsaw University of Technology, Noakowskiego 3, 00-664 Warsaw, Poland; aostrowski@ch.pw.edu.pl (A.O.); aldona@ch.pw.edu.pl (A.Z.); 3 Department of Analytical Chemistry and Biomaterials, Faculty of Pharmacy, Medical University of Warsaw, Banacha 1, 02-097 Warsaw, Poland; mpisklak@wum.edu.pl; 4 International Institute of Molecular and Cell Biology in Warsaw, Księcia Trojdena 4, 02-109 Warsaw, Poland; kskowronek@iimcb.gov.pl (K.S.); mwiweger@iimcb.gov.pl (M.W.)

**Keywords:** caffeine, cyclodextrins, mechanochemical synthesis, analytical methods, toxicity, zebrafish

## Abstract

Mechanochemical and in-solution synthesis of caffeine complexes with α-, β-, and γ-cyclodextrins was optimized. It was found that short-duration, low-energy cogrinding, and evaporation (instead of freeze-drying) are effective methods for the formation and isolation of these complexes. The products obtained, their pure components, and their mixtures were examined by powder X-ray diffraction (PXRD), differential scanning calorimetry (DSC), FT-IR and Raman spectroscopy. Moreover, molecular modeling provided an improved understanding of the association process between the guest and host molecules in these complexes. The complexes were found to exhibit high toxicity in zebrafish (*Danio rerio*) embryos, in contrast to pure caffeine and cyclodextrins at the same molar concentrations. HPLC measurements of the caffeine levels in zebrafish embryos showed that the observed cytotoxicity is not caused by an increased caffeine concentration in the body of the organism, as the concentrations are similar regardless of the administered caffeine form. Therefore, the observed high toxicity could be the result of the synergistic effect of caffeine and cyclodextrins.

## 1. Introduction

Caffeine (Caf) is a purine alkaloid with a number of beneficial effects. For centuries, humans have used the properties of caffeine to improve cognitive abilities, originally by chewing coffee berries and today by drinking coffee bean infusions and caffeinated drinks. Caffeine also has a therapeutic effect on various metabolic diseases and obesity [1,2]. Because it is a small and nonpolar molecule, caffeine is ideally suited for complexing with cyclodextrins (CDs).

CDs are cyclic oligosaccharides composed of glucose monomers. Depending on the number of subunits (six, seven or eight), we can distinguish α-cyclodextrin (α-CD), β-cyclodextrin (β-CD), and γ-cyclodextrin (γ-CD), respectively. The specific spatial structures (a truncated cone with a centrally situated cavity) of these compounds enable the formation of inclusion complexes with small weakly polar molecules [3]. This process results in the protection of the guest molecule against external factors (humidity, light, and heat) and much better solubility in water. Complexing also removes the taste and smell of the substance and increases the bioavailability of guest molecules [4,5,6].

The complexation of caffeine by native CDs (excluding γ-CD) in solution was investigated using physicochemical methods [7,8,9]. The experimental methods employed focus on various spectroscopic techniques: fluorimetry [7], UV-vis [8], and NMR [9]. Although the interactions between caffeine and CDs have been thoroughly examined in solution, the solid-state complexes of caffeine with CDs have not been successfully synthetized yet in our opinion. There are only two reports in the literature describing the attempts to obtain solid complexes of caffeine with α-CD [10] and β-CD [10,11]. However, the reported synthetic protocols and analytical proofs raised our concerns. Putative caffeine complexes with β-CD were attempted to be prepared by cogrinding, kneading, and colyophilization methods using non-stoichiometric quantities of reagents (1g per 1g) [11]. Notably, no attempts to isolate the products were made. In the cited [10] solid caffeine complexes with both α-CD and β-CD were reportedly synthesized by coprecipitation. However, only one analytical method (FT-IR spectroscopy) was employed to confirm complexation. In our opinion, the attached FT-IR spectra were inconclusive to claim the formation of true complexes.

The aim of the current study was to optimize the synthesis of solid caffeine complexes with all three native (natural) CDs by mechanochemical and in-solution methods. To confirm the formation of complexes, a number of analytical methods, including powder X-ray diffraction (PXRD), differential scanning calorimetry (DSC), and Fourier transform infrared (FT-IR) and Raman spectroscopy, were employed.

**Figure 1:**
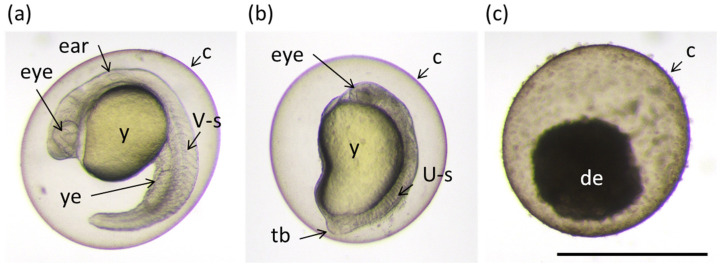
Examples of different phenotypes of zebrafish embryos at 24 h post fertilization (hpf) found in the bioassay described in the Section 2.6 “Toxicity”. (**a**) Normally developed embryo; (**b**) embryo with underdeveloped eye, brain and impaired extensions of yolk sack and tail; and (**c**) dead embryo. Ear; eye; y: yolk; ye: yolk extension; V-s: v-shaped somite; U-s: abnormally-shaped somite; tb: tail bud; and de: decomposing embryo. Scale bar equals 1 mm.

The caffeine-cyclodextrin complexes, apart from physicochemical and in silico analysis, served for verification of the following hypothesis. It is well known that CD complexes show increased permeability through biological membranes in comparison to sole guest molecules [12,13,14,15]. Therefore, we suspected that complexes could reveal higher bioactivity than uncomplexed caffeine. Fortunately, several tests have been established to examine biological functions in vitro or in vivo. Among them, the “Fish Embryo Acute Toxicity (FET) Test” is often used to access the acute or lethal toxicity of chemical species in vertebrates. Commonly, FET is performed on embryonic stages of a small tropical fish, namely zebrafish (*Danio rerio*; Figure 1).

Zebrafish shares over 70% genetic identity and high conservation of developmental and molecular processes with humans [16,17]. In addition, experiments which are performed at early stages of zebrafish development (up to 120 h post fertilization (hpf)) when embryos and larvae are incapable of independent feeding, do not require permit from the ethics committee. Zebrafish model was already used for studies on the effect of caffeine on, e.g., behavior [18] and development [19]. Although some studies demonstrated the attenuation of biological functions in zebrafish by some CDs (for example [20] and [21]) we are the first to report increased toxicity of caffeine when complexed with CDs. 

## 2. Results and Discussion

### 2.1. Synthesis

Solid CD complexes of caffeine (Caf@CDs) were prepared either by cogrinding of equimolar mixtures for 30 min in a mortar with a pestle in the absence (1) or presence (2) of a few drops of water, or (3) by evaporation-induced deposition from equimolar aqueous solutions. In route 3, a rotatory evaporator was used, which is a much simpler approach than the usual freeze-drying method. According to the literature [22,23], 30 min of cogrinding can be a sufficient time to obtain CD complexes in some cases. Prior to cogrinding, CDs were dried because those substances are supplied with a high content of water (up to a dozen percent), which can lead to invalid estimation of molar ratios. It is worth noting that drying natural CDs before usage is neglected in many published studies.

For the sake of clarity, the names of the appropriate Caf@CDs are followed by the numbers 1, 2, and 3, which refer to the route of synthesis. To indicate that Caf@CDs complexes were in fact formed, counterpart mixtures (Caf+CDs) were also prepared and subjected to the same physicochemical measurements. The abbreviations of the mixtures obtained from anhydrous CDs end with the number 1 (e.g., Caf+β-CD1). On the other hand, mixtures prepared from aqueous solutions are followed by the number 3 (e.g., Caf+γ-CD3). The pure samples obtained from evaporation-induced deposition were also denoted with the number 3 (e.g., Caf3). The abbreviations of all the substances under investigation (single components, mixtures, and putative complexes) are collected in Table 1.

### 2.2. Powder X-ray Diffraction (PXRD)

**Figure 2:**
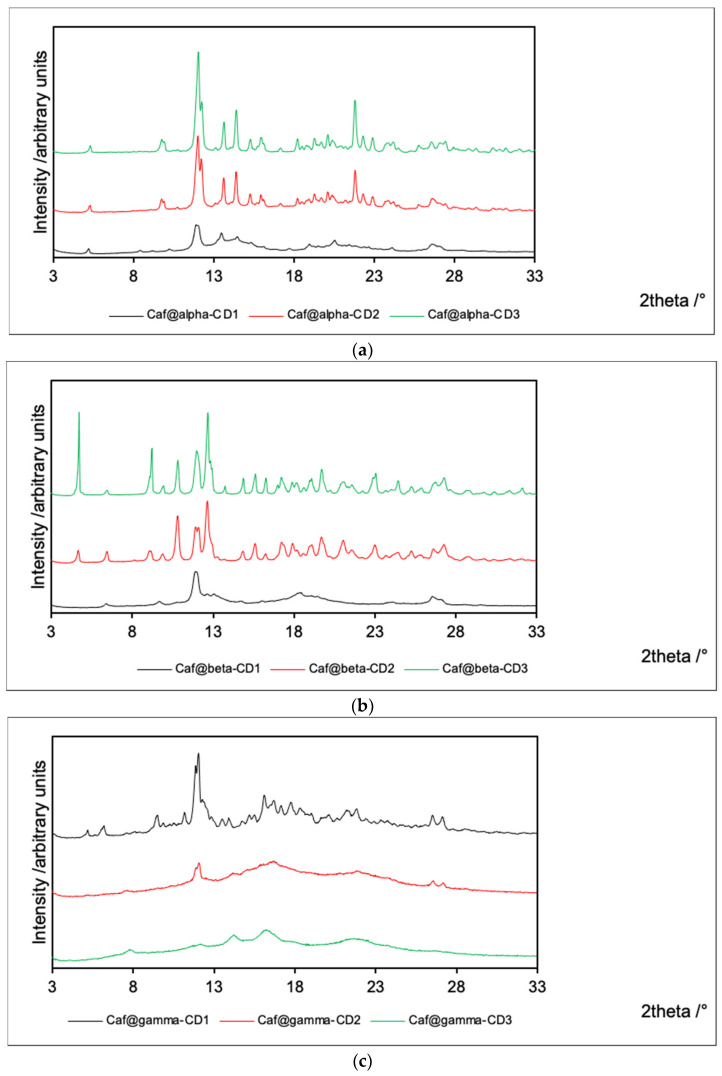
PXRD patterns for Caf@CD obtained by three methods (1: cogrinding; 2: cogrinding with the addition of water drops; and 3: depositing from soln.). (**a**) α-CD; (**b**) β-CD; and (**c**) γ-CD series.

The PXRD patterns were recorded for all the chemical species under investigation (Table 1) and are collected in Figure 2 and Supplemenatry Figures S1–S3. To facilitate the comparison of the results, the PXRD patterns for the putative complexes obtained by each method are grouped for each CD series in Figure 2, while mixtures and counterpart complexes are depicted together with their pure components in Supplementary Figures S1–S3.

In general, the signals for mixtures obtained from both dried and evaporated ingredients are a simple sum of the peaks collected for single components, as expected. However, the recording of the PXRD patterns for both mixtures and putative complexes is a crucial approach to demonstrate the complexation process. According to the literature [24,25,26], CD complexes with active pharmaceutical ingredients (APIs) are usually amorphous, in contrast to their counterpart mixtures, which are usually crystalline.

The PXRD patterns obtained for the α-CD and β-CD series of products are similar. The products obtained by cogrinding without the addition of water exhibit low crystallinity. On the other hand, both products from cogrinding in the presence of water and from evaporation of the aqueous solution exhibit high crystallinity. In the latter two cases, the PXRD patterns are similar to those of analogous mixtures. Therefore, one can conclude that water disturbs the association between caffeine and α-CD or β-CD.

The opposite situation is observed for γ-CD complexes. The flat PXRD patterns for both Caf@γ-CD2 and Caf@γ-CD3 indicate their amorphous character. On the other hand, Caf@γ-CD1 is crystalline, and its PXRD pattern is similar to that of its counterpart mixture (Caf+γ-CD1). Therefore, the presence of water seems to be an essential condition for the complexation of caffeine with γ-CD. Moreover, the PXRD pattern of Caf+γ-CD1 contains signals from hydrated γ-CD3 instead of dry γ-CD1 (Supplementary Figure S3). Therefore, it is plausible that this sample contained moisture adsorbed from the air. This observation could additionally lead to the conclusion that the presence of water is thermodynamically favored in the case of γ-CD complexes. The supporting role of water as a space-filling molecule in inclusion complexes of CDs has been reported in many crystal structures (derived from single-crystal X-ray diffraction refinements) of such systems [27,28,29].

### 2.3. Differential Scanning Calorimetry (DSC)

DSC thermograms were registered for single components, mixtures, and putative complexes and are presented in Supplementary Figures S4–S18. Temperatures of the peaks and the respective heat values are collected in Table 2. The descriptions of the DSC curves for pure ingredients and their mixtures can be found in the Supplementary Material.

In general, the thermograms of mixtures prepared from evaporated ingredients (Caf+CD3) are, in a majority of cases, equivalent to the sum of the signals observed for the pure components. On the other hand, the thermograms of mixtures obtained from dried CDs (Caf+CD1) show new signals that could have come from newly formed phases. However, the PXRD measurements preclude the possibility of the formation of new phases in all the mixtures under investigation. The anomalous DSC curves for Caf+CD1 may be due to the energy absorbed by the samples during DSC scans [30,31]. Nevertheless, in the presence of water, the supplied heat did not influence the thermal behavior of such mixtures, in contrast to anhydrous mixtures.

The superimposed thermograms for Caf+α-CD1 and Caf@α-CD1 (Supplementary Figure S5) reveal clear similarities, which are especially apparent for the transition at 230 °C corresponding to pure Caf1. There is a very distinct endothermic peak of similar height for both Caf+α-CD1 and Caf@α-CD1 (ca. 23–26 J⋅g^−1^). However, the intensity of this peak is considerably lower than that for pure Caf1. In both cases, the endothermic peak is preceded by a small exothermic peak. The signal from α-CD1 can be seen for both Caf+α-CD1 and Caf@α-CD1, though it is shifted toward lower temperatures. It is clear that this process involves several phases. In the case of Caf+α-CD1, the more intense transition is placed at 130 °C, with a sharp maximum at 152 °C. Whereas for Caf@α-CD1, this process starts at 100 °C, and the corresponding peak is very broad and without a sharp maximum. There is a new exothermic peak in the thermogram for Caf@α-CD1 (203 °C, 11 J⋅g^−1^). This peak is not observed in the thermograms for the pure ingredients (Caf1 and α-CD1), nor in that for the corresponding mixture (Caf+α-CD1). Therefore, this phenomenon can be related to the formation of a true complex (Caf@α-CD1).

The DSC thermograms for Caf+α-CD3 and Caf@α-CD3 (Supplementary Figure S7) are roughly similar, with small deviations in the endothermic peak onsets. In the case of Caf@α-CD3, the peak associated with the melting of Caf3 is significantly shifted from 235 to 225 °C. Moreover, a new peak appears at 250 °C, which is followed by decomposition of the whole system.

Comparison of the thermograms for three putative complexes in the α-CD series (Supplementary Figure S8) leads to important conclusions that are consistent with the PXRD results. The thermograms for both Caf@α-CD2 and Caf@α-CD3 are very similar to each other, with peaks from the pure ingredients at similar temperatures and of comparable intensities in those thermograms. Different features are apparent in the thermogram obtained from Caf@α-CD1. Its thermogram did not reveal peaks from pure substances. Furthermore, only in this case was the exothermic peak at 203 °C detected, being associated with a small heat effect. Therefore, the formation of a complex between caffeine and α-CD prepared by cogrinding without the addition of water (Caf@α-CD1) was confirmed. On the other hand, cogrinding in the presence of water, as well as the reaction in solution, failed to yield caffeine complexes with α-CD.

In the case of Caf+β-CD1 and Caf@β-CD1, the DSC curves are similar (Supplementary Figure S10). There is a broad peak with the onset at 70 °C, probably attributable to pure β-CD1, on the thermograms for both the mixture and the putative complex. However, this peak is shifted toward lower temperatures. In the case of Caf+β-CD1, this peak exhibits a clear maximum and is sharper than the corresponding peak for the putative complex. The heats connected with the process discussed above are comparable for both substances. The endothermic peak coming from pure Caf1 is found for Caf+β-CD1 and Caf@β-CD1. Nevertheless, its intensity decreases in comparison with that of the pure ingredient (Caf1). In the case of Caf@β-CD1, a new exothermic peak appears at 208 °C, with a small heat effect (13 J⋅g^−1^). Notably, this peak is not observed for the mixture or pure components.

The DSC curves for Caf+β-CD3 and Caf@β-CD3 (Supplementary Figure S12) exhibit the following differences. In the case of the mixture, a broad peak at 141 °C, with two prominent maxima, is present. The heat of the process associated with this peak is 479 J⋅g^−1^. On the other hand, two noticeable peaks are formed for the putative complex. One of them is broad and reaches a maximum at 152 °C, whereas the other is sharp and reaches a maximum at 182 °C (250 J⋅g^−1^). Moreover, a small peak at 232 °C, produced by pure Caf3, can be seen in the thermograms for Caf+β-CD3 and Caf@β-CD3. Its intensity is 200 times lower than that for Caf3.

The thermograms for Caf@β-CD1, Caf@β-CD2, and Caf@β-CD3 (Supplementary Figure S13) exhibit analogous differences as in the case of putative complexes in the α-CD series. That is, the thermograms for Caf@β-CD2 and Caf@β-CD3 are very similar. In both cases, three endothermic peaks with onsets at similar temperatures and small intensity deviations are visible. On the other hand, the thermogram for Caf@β-CD1 is different from the thermograms for the other putative complexes in the β-CD series. In the case of Caf@β-CD1, a broad endothermic peak of small intensity can be seen in the range of 70–170 °C and also two exothermic peaks at 208 and 225 °C. It is worth noting that an endothermic peak at 250 °C was formed only in the DSC curve for Caf@β-CD1. Therefore, it seems justified to claim that caffeine forms complexes with β-CD only by cogrinding in the absence of water (Caf@β-CD1).

There are only two endothermic peaks in the thermograms for Caf+γ-CD1 and Caf@γ-CD1 (Supplementary Figure S15). The peak at 166 °C can be seen for both substances. However, this peak is more intense for the mixture (89 J⋅g^−1^) than for the putative complex (12 J⋅g^−1^). In the case of Caf@γ-CD1, a new peak with the onset at 205 °C (106 J⋅g^−1^) is registered. This peak is sharp and intense, and it could be a peak from pure γ-CD1. However, it is shifted towards lower temperatures by 15 °C with respect to the pure substance.

The DSC curve for Caf@γ-CD3 exhibits only one peak at 168 °C, which is also observed for Caf+γ-CD3 (Supplementary Figure S17). The intensity of this endothermic peak for the putative complex is approximately one-half of that of the mixture. In the case of this mixture, there are also peaks coming from pure γ-CD3. The peak from pure Caf3 is absent for both Caf@γ-CD3 and Caf+γ-CD3.

Similar DSC curves were registered for caffeine complexes with γ-CD obtained by the three methods (Supplementary Figure S18). In each of those thermograms, one large endothermic peak of similar intensity and different onset temperatures is observed. In the case of Caf@γ-CD1, that peak is placed at the highest temperature (205 °C) and is significantly sharper than for other complexes. This peak is observed at similar temperatures (178 and 168 °C) for Caf@γ-CD2 and Caf@γ-CD3, respectively.

As concerns putative complexes in the γ-CD series, in view of the abovementioned facts the DSC measurements can be interpreted as supporting the results obtained from PXRD experiments in which caffeine forms complexes with γ-CD (Caf@γ-CD2 and Caf@γ-CD3) only by methods in which water is present.

### 2.4. Vibrational Spectroscopy

**Figure 3:**
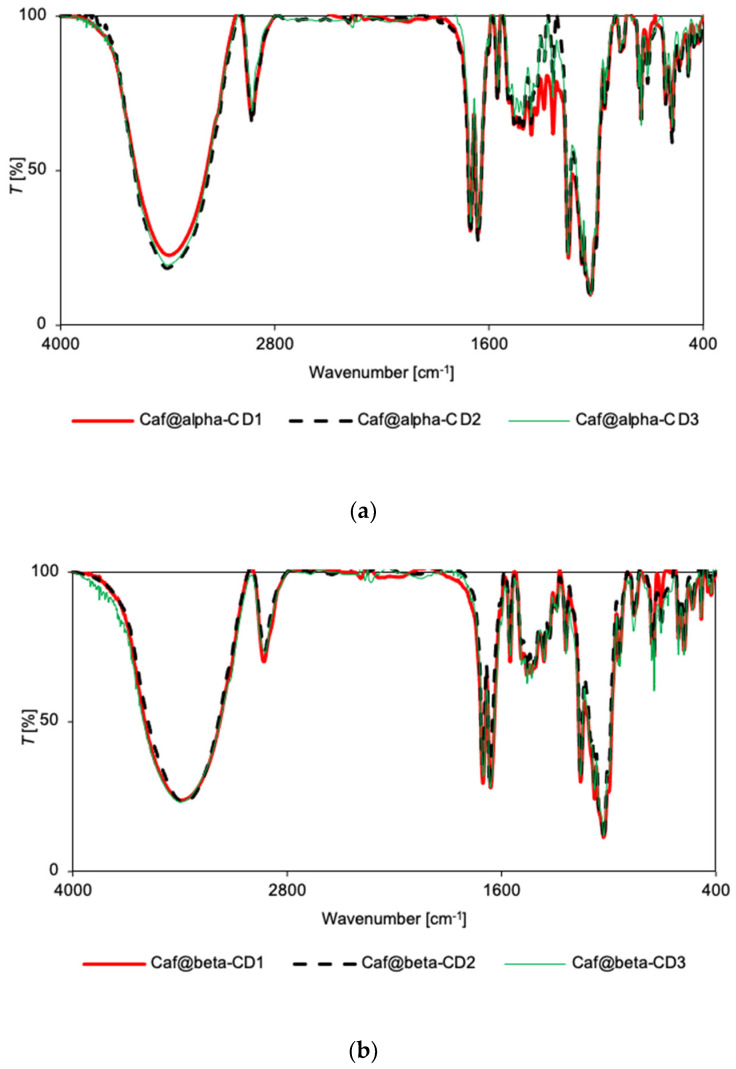

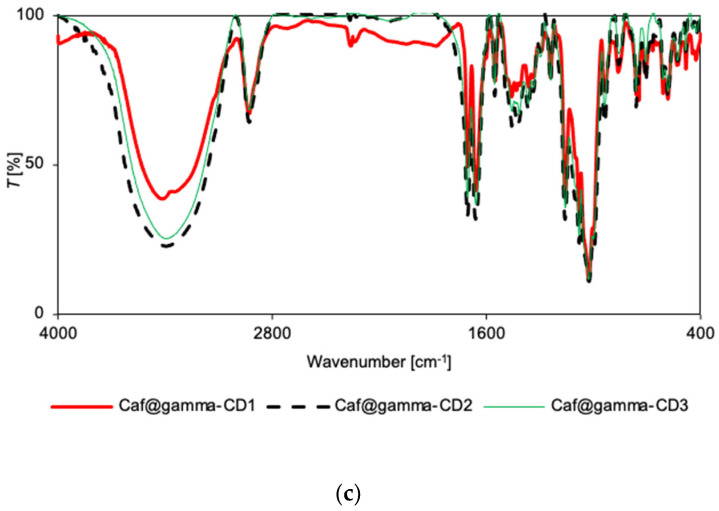
FT-IR spectra for Caf@CD obtained by three methods (1: cogrinding; 2: cogrinding with the addition of water drops; and 3: depositing from soln.). (**a**) α-CD; (**b**) β-CD; and (**c**) γ-CD series.

There were no significant differences in the relative intensity or displacement of the bands in the IR spectra of the mixtures and corresponding putative complexes for the α-CD series (Supplementary Figures S19 and S20). Only in the case of Caf@α-CD1 did a small broadening of bands occur (in the range 1200–1800 cm^−1^), suggesting some weak interactions between the guest molecule and α-CD that could induce greater disorder (and thus the loss of crystallinity). Nevertheless, the IR spectra for all three putative complexes of α-CD are very similar (Figure 3a), with the only clear difference being a broadening of bands in the range of 400–1500 cm^−1^ for Caf@α-CD1. So, there is no evidence of hydrogen bond formation, although it could be expected in this system as the caffeine molecule has three potential acceptor sites: two carbonyl groups and an imidazole ring nitrogen. However, no changes could be seen either in the carbonyl stretching band position or intensity, or in the imidazole ring vibrations.

**Figure 4:**
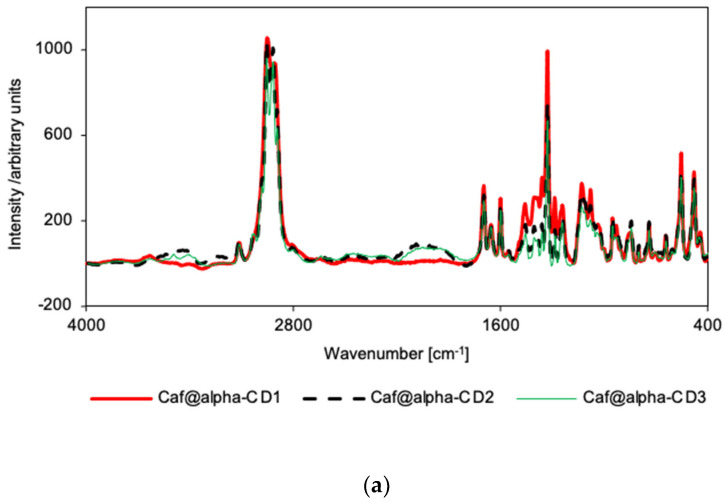

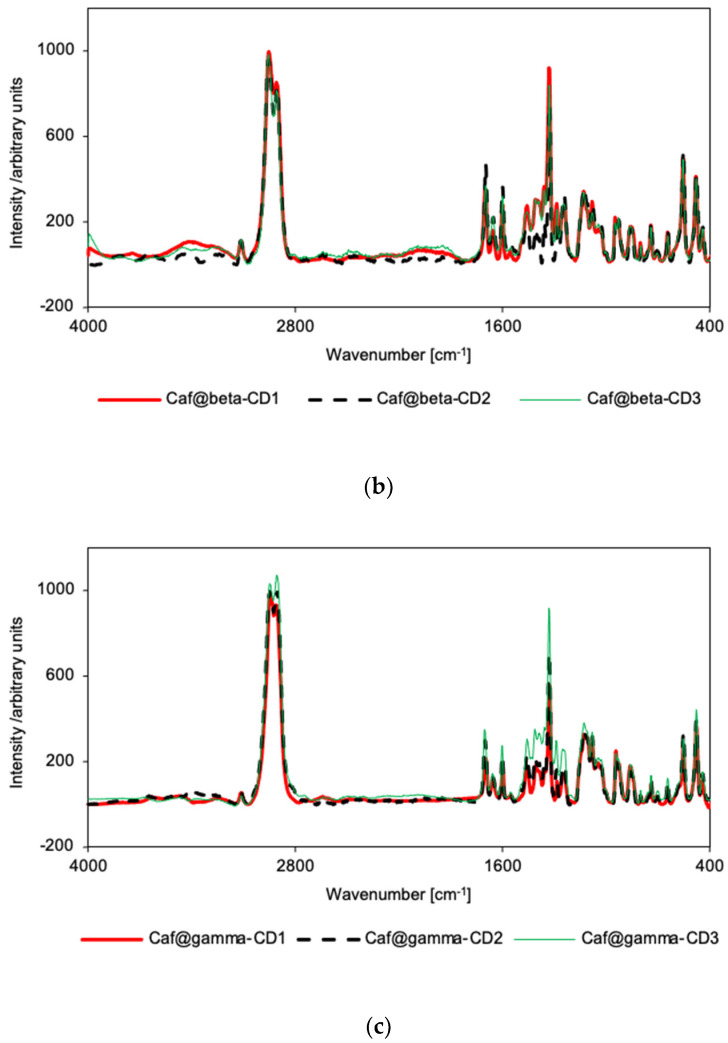
Raman spectra for Caf@CD obtained by three methods (1: cogrinding; 2: cogrinding with the addition of water drops; and 3: depositing from soln.). (**a**) α-CD; (**b**) β-CD; and (**c**) γ-CD series.

Similarly, in the Raman spectra for the putative complexes, a broadening of α-CD bands can be seen, as compared with counterpart mixtures (Supplementary Figures S25 and S26), especially in the range of 1200–1800 cm^−1^. The broadening is more prominent in the case of Caf@α-CD1 for the CD bands in the range 1200–1530 cm^−1^ (Figure 4a). This result confirms the decrease in crystallinity of the sample. Additionally, in the Raman spectrum for Caf@α-CD3, as compared with the corresponding mixture spectrum, the decreases in relative intensities of the following bands indicate the restriction of these vibrations by the restricted space inside α-CD: the 554 cm^−1^ band, ascribed to the caffeine breathing mode [32]; the 739 cm^−1^ band, ascribed to the out-of-plane bending of both caffeine rings; and the 1327 cm^−1^ band, ascribed to the *ν*_ring_(imidazole) + *ν*_ring_(pyrimidine) mode (Supplementary Figure S26).

In the IR spectra for the putative complexes and mixtures in the β-CD series, the spectral patterns for Caf@β-CD3 and Caf+β-CD3 (Supplementary Figure S22) are nearly identical, while for Caf@β-CD1 and Caf+β-CD1 (Supplementary Figure S21) the differences in intensities of some bands could be seen. Namely, in the IR spectrum of Caf@β-CD1 there is an increase of intensity of the bands at 1674 and 1687 cm^−1^, corresponding to C=O stretching, and at 1547 cm^−1^, arising from *δ*(HCN) + *ν*_ring_(imidazole) + *ν*_ring_(pyrimidine) mode, as well as a decrease of intensity of the band at 609 cm^−1^, ascribed to out-of-plane bending of the imidazole ring, as compared with the spectrum of a physical mixture. The small increase of relative intensity of C=O stretching bands could suggest a hydrogen bond formation with caffeine carbonyl groups acting as hydrogen bond acceptors. Still, no frequency shift was observed, suggesting a rather weak interaction. As for the 609 cm^−1^ band, it overlaps with the CD band, so the difference could be due to either this overlap or the removal of hydration water, as it was shown that the interaction between caffeine and water is through a weak hydrogen bond with imide nitrogen serving as an acceptor [33].

However, when the IR spectra for all the putative complexes for the β-CD series are compared (Figure 3b), they show the same pattern with the exception of slightly higher intensities of the 611, 746, and 857 cm^−1^ bands in the case of Caf@β-CD3. As these bands were ascribed to the vibrations of the imidazole ring [32] it is possible that the presence of water is unfavorable for the introduction of this part of the caffeine molecule into the β-CD cavity. On the other hand, in the Raman spectra there are no differences between the relative intensities of the spectra for all the putative complexes (Figure 4b), or between the spectra of the putative complexes (Caf@β-CD1 and Caf@β-CD3) and the corresponding mixtures (Caf+β-CD1 and Caf+β-CD3) (Supplementary Figures S27 and S28). Nevertheless, in the presence of water (Caf@β-CD3), a slight broadening of the caffeine bands in the range of 1500–1800 cm^−1^ (the *ν*(C=O) and *ν*(C=C) + *δ*(HCN) modes) (Figure 4b and Supplementary Figure S28) can be seen in the Raman spectra, which may indicate weak interactions of these groups with the host molecule.

For the γ-CD series, no differences in the spectral patterns were observed for Caf@γ-CD1 and Caf+γ-CD1, nor for Caf@γ-CD3 and Caf+γ-CD3 in either the IR or Raman spectra (Supplementary Figures S23, S24, S29 and S30). Thus, the differences observed in the IR and Raman spectra of the putative complexes prepared by various methods (Figure 3c and Figure 4c), namely, the broadening of bands in the range of 1200–1530 cm^−1^ for Caf@γ-CD3, are probably due to the presence or absence of water in the system and not to the formation of new bonds between the guest and host molecules.

Vibrational spectroscopy supported the findings from PXRD and DSC analyses that solid caffeine complexes with α-CD and β-CD can be obtained by cogrinding in the absence of water, although there are probably no strong interactions between the host and guest molecules. The other synthetic methods studied appear to have been unsuccessful. FT-IR and Raman spectroscopy failed to confirm the complexing properties of γ-CD. Similar results, in which FT-IR spectroscopy failed to prove the formation of true solid CD complexes with APIs, while it was confirmed by other analytical techniques, were reported earlier [24,34,35].

### 2.5. Molecular Modeling

Molecular modeling can be used as a tool to characterize the binding behavior between CD as hosts and small organic molecules as guests. The computational protocol used in this work was largely inspired by a number of previously reported studies [36,37,38,39,40]. In all of them, as well as in many other published works, the density functional theory (DFT) method was shown to be a powerful tool for studying CD complexes, combining high computational efficiency with the required accuracy.

Recently, Oqmhula et al. [40] have shown that the application of B3LYP DFT functional with Grimme’s dispersion correction can provide results comparable with those obtained by significantly more demanding Diffusion Monte Carlo (DMC) calculations. 

**Figure 5:**
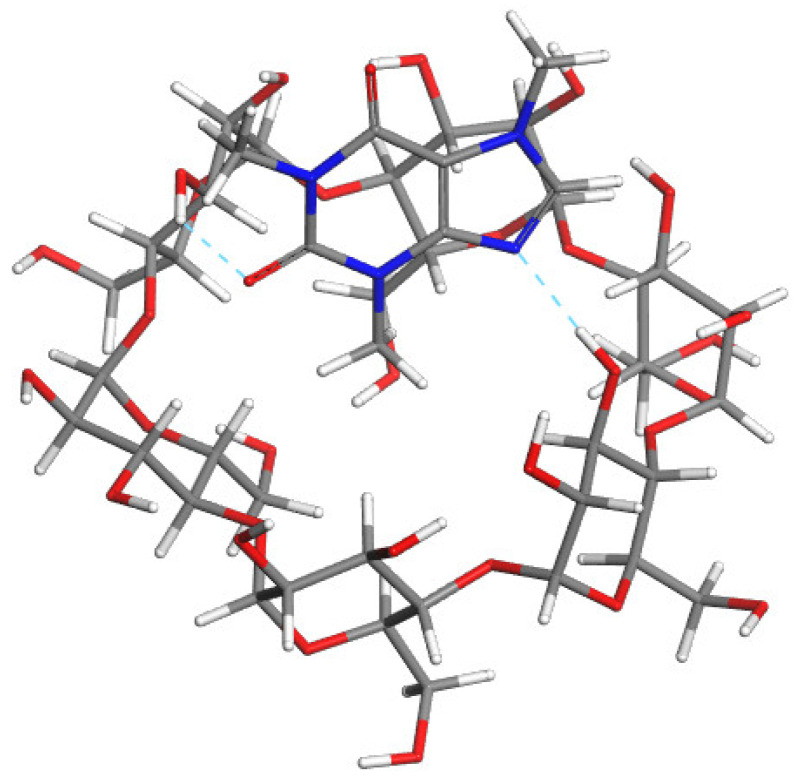

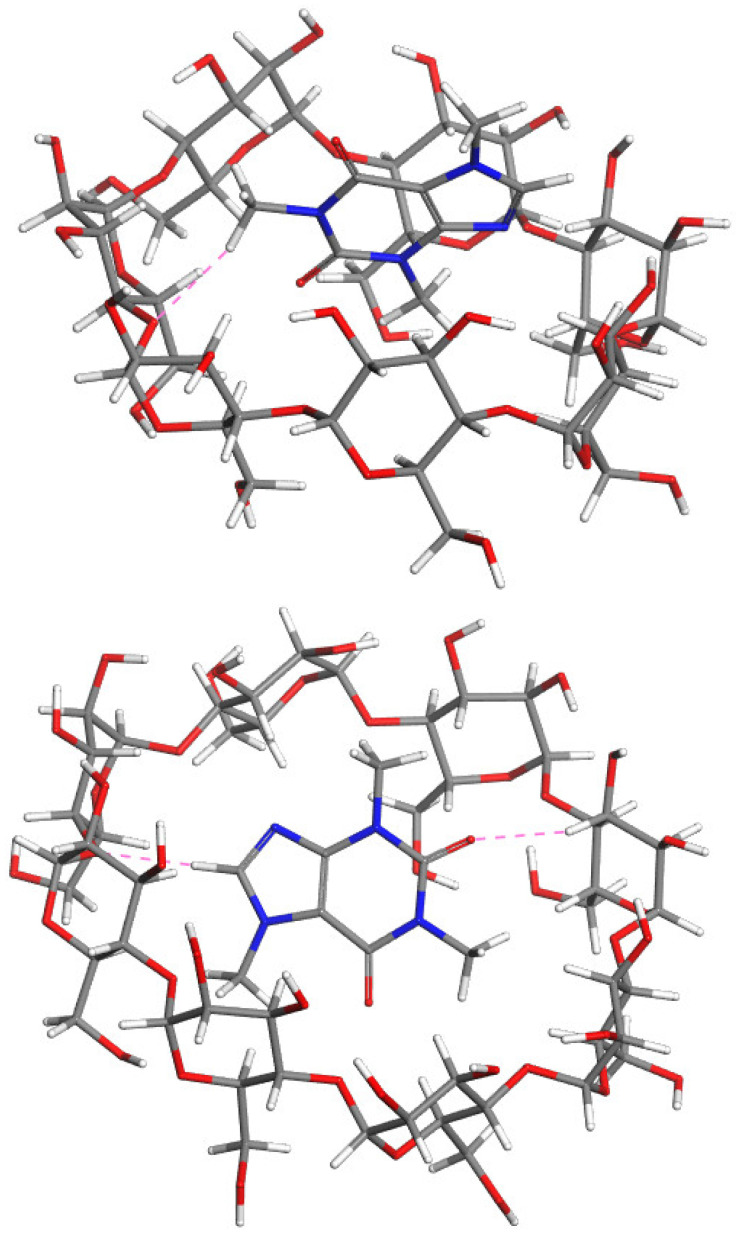
The most stable modeled structures: Caf@α-CD (upper); Caf@β-CD (middle); and Caf@γ-CD (lower). Atoms: C (grey); H (white); O (red); and N (blue). The cyan and pink dashed lines represent possible hydrogen bonds and close contacts stabilizing the complexes, respectively.

As explained in detail in Section 3.7 “Molecular modeling”, the calculations were performed in two stages. First the molecular docking was applied to effectively generate the low energy poses. Then the complexes of the lowest energy were optimized at the DFT level. The second stage was done both with and without the application of the polarizable continuum model (PCM) solvation scheme to determine the effect of water on the structure and energy of the complexes. The results of the molecular modeling calculations are presented in Table 3 and Figure 5. A more negative binding energy, ∆*E*_bind_, indicates a more energetically favorable complexation. The ∆*E*_bind_ values were found to be negative for all the studied complexes and orientations, both with (∆*E*_bind_/PCM) and without the application of PCM solvation scheme.

In the case of Caf@α-CD, the most stable conformation was the one in which the caffeine molecule was situated close to the wider rim of the CD molecule. That orientation allowed the formation of two H-bonds between the host and the guest molecules, namely between N9 and O2 as acceptors and H atoms forming hydroxyl groups of C4 atoms of α-CD as donors. Of the orientations obtained by molecular docking those in which the caffeine was oriented perpendicularly to the α-CD plane were found to be less energetically favorable, due to the steric hindrance. 

The orientation of caffeine in the structure of Caf@β-CD was found to be similar to the one in Caf@α-CD. Again, the caffeine molecule was situated close to the wider rim of the CD molecule, with the methyl group at N3 oriented inside and the other two methyl groups oriented outside. However, in this complex the larger size of the CD cavity enabled the caffeine molecule to penetrate deeper, which was forced by the attraction between the nonpolar caffeine molecule and hydrophobic cavity of β-CD.

For both Caf@α-CD and Caf@β-CD the energies of the solvated complexes were found to be less negative than those of their nonsolvated counterparts. This is in agreement with the experimental PXRD observation that water disturbs the association between caffeine and α-CD or β-CD.

A different orientation of the guest molecule was obtained for Caf@γ-CD. Due to the increased size of the γ-CD cavity in comparison with α-CD and β-CD it was possible for the caffeine molecule to hide inside the hydrophobic cavity and form the true inclusion complex. Caffeine was found to be oriented transverse to the γ-CD and in that orientation none of the methyl groups of caffeine protruded from the γ-CD. In the case of this CD, the energy of the solvated complex was found to be lower than that of its nonsolvated counterpart. This nicely corresponds with the conclusions based on the PXRD analysis results stating that the presence of water is thermodynamically favored in the case of γ-CD complexes.

### 2.6. Toxicity

**Figure 6:**
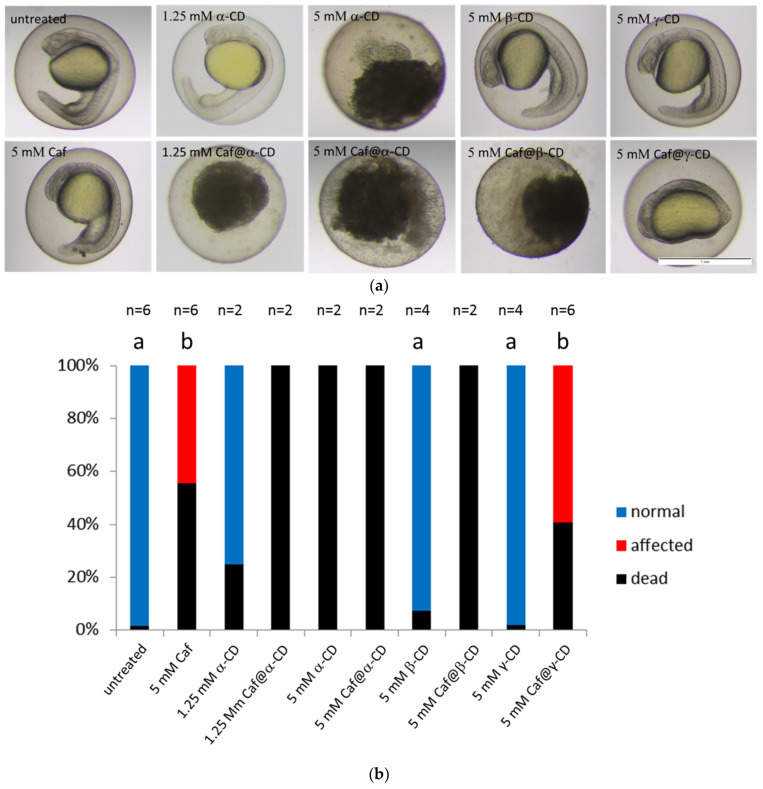
The effect of Caf@CDs on early development of the zebrafish embryo. (**a**) Images of zebrafish embryos after exposure to caffeine (Caf), CDs or Caf@CDs from 4 till 24 hpf. Embryos exposed to Caf@γ-CD show underdeveloped eyes, ears and severely impaired convergent extension. Less severe morphological abnormalities were also present in embryos exposed to 5 mM Caf. Exposure to 5 mM α-CD, 1.25 mM Caf@α-CD, or 5 mM Caf@β-CD was lethal to the embryos. Examples of live embryos at a most advanced developmental stage are shown. Scale bar, 1 mm. (**b**) Changes in the proportion between the dead, morphologically abnormal (affected), and unaffected (normal) embryos. The percentage of embryos which died upon treatment with Caf or Caf@CDs was significantly higher than that of untreated control. Also, pure α-CD was more toxic than the other two CDs, whereas β-CD and γ-CD had no effect on both the fish survival and their morphology. In order to reduce the number of animals, in the case of treatments which caused 100% rapid mortality, the experiments were repeated twice. Bars present mean values, n is the number of repetitions, each on 24 embryos. Mann-Whitney U test was performed on samples with ≥3 biological replicas. Statistically significant differences (*p* < 0.01) were marked with different letters.

The zebrafish model was used to assay the toxicity of various chemical species. Lantz-McPeak et al. [19] demonstrated the dose-dependent effect of caffeine on zebrafish embryo development, finding an approximately 50% reduction in embryo length upon treatment from 28 h post fertilization (hpf) for 24 h (the time covering early to late pharyngula period) with 5 mM caffeine. We found that zebrafish embryos treated for 24 h with the same concentration of caffeine at a later time (from 48 to 72 hpf) also showed similar reduction of body length as described by Lantz-McPeak et al. [19] (Supplementary Figure S31) whereas the treatment starting at 4 hpf (mid blastula stage) had an even more severe effect, i.e., it caused high mortality and severe malformations in all surviving embryos (Figure 6 and Supplementary Figure S31). In all surviving embryos which were exposed to caffeine from 4 hpf, less transparent bodies, underdeveloped eyes and brain, impaired tail and yolk sac extension as well as abnormally shaped somites were observed at 24 hpf (Figure 6 and Supplementary Figure S31).

On the other hand, limited information is available as concerns the effect of CDs on the zebrafish development. It was shown that 5 mM concentration of methyl-β-cyclodextrin (Me-β-CD) was sufficient to cause abnormal cytokinesis, whereas 2-hydroxypropyl-β-cyclodextrin (HP-β-CD) had no effect on zebrafish embryo development when used at a concentration of 1% or 2 μM, respectively [20,41,42]. To our knowledge, no data are available showing the impact of α-CD, β-CD, and γ-CD on the zebrafish development. That is why we also tested the toxicity of pure CDs using our zebrafish model. We found that up to 5 mM concentrations of γ-CD did not affect embryo development irrespectively of the stage at which fish were treated (Figure 6 and Supplementary Figure S31). Moreover, the treatment with 5 mM β-CD had no effect on embryo development whereas 5 mM of α-CD caused 100% mortality (Figure 6). The concentration of α-CD had to be reduced to 1.25 mM, so as not to cause the teratogenic effect (Figure 6).

As the complexation with CDs is known to attenuate the biological function of APIs, we tested the effect of caffeine when complexed with three native CDs and compared it with the toxicity of the pure components. Caf@CDs exhibited a more pronounced effect than caffeine or CDs alone. 5 mM Caf@γ-CD dramatically impaired convergent extension resulting in embryos with severely affected morphology, whereas 5 mM Caf@β-CD or 1.25 mM Caf@α-CD caused death of the treated embryos (Figure 6). Judging by the degree of decomposition that was observed after 20 h of treatment (at 24 hpf, Figure 6), the embryos which were exposed to 5 mM α-CD, ≥1.25 mM Caf@α-CD, and 5 mM Caf@β-CD, died soon after the treatment started. The opposite observation was reported by Du et al. [43] who used complexing with β-CD to lower the toxicity of fluorescent CdTe nanocrystals. Moreover, Geng et al. [44] demonstrated that complexation with HP-β-CD significantly reduced the toxicity of butachlor to fish, at the same time boosting mobility and activity of this herbicide. However, Radi et al. [42] found that HP-β-CD in complex with one of the tested compounds showed antiangiogenic activity. Hence, it is possible that the biological properties of CD complexes highly depend on the API, and that is why the toxicity of Caf@β-CD was so different from that of CdTe@β-CD. 

### 2.7. Caffeine Levels in the Zebrafish Embryos

CDs are known to affect the chemical, physical, and thermal stability of drugs as well as their bioavailability, including the increased dissolution rate and solubility of drugs due to complex formation [45]. As fish were treated with completely dissolved substances, it is unlikely that the increased toxicity of Caf@CDs was caused by the greater solubility of caffeine. However, complexing might have affected the bioavailability of caffeine, e.g., by reducing the hydrophobicity of caffeine and therefore increasing its absorption. To verify this, high-performance liquid chromatography (HPLC) was used to measure the amount of caffeine in the zebrafish embryos and larvae that had been exposed to caffeine or Caf@CDs for 0, 5, 10, 15, 30, 45, and 60 min and 24 h. Similar profiles were obtained for all the tested compounds (caffeine, Caf@α-CD, Caf@β-CD, and Caf@γ-CD; Supplementary Figure S32a). Measurements labeled as 0 min exposure (quick submersion in the tested solution followed by three washes with E3 medium) failed to quantify caffeine due to a very low signal, indicating that our assay indeed corresponds to caffeine in the zebrafish body and is not affected by the compound bound to the skin of zebrafish embryos or larvae. The uptake of caffeine was quick. After 5 min of exposure, caffeine was readily detected. Caffeine delivered as Caf@γ-CD showed slightly greater accumulation in the first 15 min of exposure than pure caffeine or Caf@α-CD and Caf@β-CD (Supplementary Figure S32b). This small difference in the uptake of the tested compounds might have been caused by prompt saturation of the system. Unfortunately, the measured levels were close to the lower limits of linearity, so we could not run those assays at lower concentrations. More sensitive methods and/or an increased number of zebrafish embryos would be required to measure the uptake of caffeine from more dilute solutions. It seems that such a small difference could be of biological importance. However, since only caffeine delivered as Caf@γ-CD showed that trend, whereas the uptake of caffeine delivered in the most toxic complex, Caf@α-CDs, had a similar profile to that of pure caffeine, it is reasonable to conclude that the differences in the uptake cannot explain the increased biotoxicity of Caf@CDs. Further studies are needed to determine whether the teratogenic effect could result from synergistic toxicity.

## 3. Materials and Methods

### 3.1. Materials

Caffeine was purchased from Sigma-Aldrich (Poznań, Poland), while α-CD, β-CD, and γ-CD were purchased from Carbosynth (Compton, UK). CDs were dried in an oven at 120 °C for 2 h. Caffeine was used as supplied, unless otherwise stated. Alternatively, caffeine and CDs were obtained as solids from evaporation of their aqueous solutions in a rotatory evaporator and were not subjected to further drying. 

### 3.2. Synthesis

Putative complexes were prepared by either cogrinding with the aid of a mortar and pestle for 30 min or evaporative deposition of aqueous solutions in a rotatory evaporator (*p* ≈ 30 mbar, *T* ≈ 60 °C). In each case, equimolar ratios of reactants were used. Cogrinding was performed either in the absence or in the presence of a few drops of water. Dried CDs, as well as their putative complexes and mixtures, were stored in a vacuum desiccator in the presence of P_2_O_5_.

### 3.3. PXRD

Laboratory PXRD patterns were recorded at room temperature on a Bruker D8 Advance diffractometer equipped with a LYNXEYE position sensitive detector using Cu-Kα radiation (*λ* = 0.15418 nm). Data were collected in the Bragg-Brentano (*θ*/*θ*) horizontal geometry (flat reflection mode) between 2° and 70° (2*θ*) during a continuous scan using 0.03° steps 960 s⋅step^−1^. The diffractometer incident beam path was equipped with a 2.5° Soller slit and a 1.14° fixed divergence slit, while the diffracted beam path was equipped with a programmable antiscatter slit (fixed at 2.20°), a Ni β-filter and a 2.5° Soller slit. Data were collected under standard laboratory conditions (temperature and relative humidity). The samples for powder diffraction were placed on a zero-background silicon wafer.

### 3.4. DSC

The phase transitions and thermal stability of the samples were studied using differential scanning calorimetry (DSC). The DSC data were obtained using a Q200 scanning calorimeter (TA Instruments) under flowing nitrogen (25 mL⋅min^−1^) at a heating rate of 10 °C⋅min^−1^ from 20 to 300 °C. The samples were placed in aluminum *T*_zero_ hermetic pans. An empty pan was used as the reference. Data analysis was carried out using the TA Universal Analysis application.

### 3.5. FT-IR

FT-IR spectra were recorded using a Perkin Elmer Spectrum 1000 FT-IR spectrometer. The transmission measurement technique was used. The samples for FT-IR analysis were prepared using the KBr tablet method. The background tablet was prepared as follows: 220 mg (±10 mg) of KBr was weighed in a cup on an analytical balance and then ground in a mortar, and a tablet was obtained using a hydraulic press (the pressing force corresponded to 10 tons). The samples of the analyzed complexes, mixtures and pure substances were prepared as follows: KBr was weighed at 220 mg (±10 mg) and then the test substance was added in an amount of 2%. The added substances had previously been ground in a mortar. For the samples of mixtures, only gentle agitation in the mortar was used, without grinding. The process of forming the tablet with the analyzed substance was the same as that used to form the background tablet. Registration of the FT-IR spectra of the prepared samples was carried out using the following parameters: wavenumber range 4000–400 cm^−1^, resolution of 2 cm^−1^, and number of scans equal to 50.

### 3.6. Raman Spectroscopy

Raman spectra were recorded using an iRaman 532 spectrometer (B&W Tek) operating with a laser emitting radiation of wavelength *λ* = 532 nm and power of 42 mW. The following measurement parameters were used: wavenumber range 4000–150 cm^−1^, resolution 4 cm^−1^, acquisition time 500 ms, number of scans 100, and laser power set at 50%.

### 3.7. Molecular Modeling

The crystal structures of guests and hosts were obtained from the Cambridge Structural Database (CSD) [46]. Caffeine, α-CD, β-CD, and γ-CD were extracted from the corresponding crystal structure files, refcodes: NIWFEE05 [47]; CHXAMH [48]; BCDEXD10 [49]; and CIWMIE10 [50] for caffeine, α-CD, β-CD, and γ-CD, respectively.

The initial structures of the studied complexes (Caf@α-CD, Caf@β-CD, and Caf@γ-CD) were obtained by molecular docking method using the Adsorption Locator program utilizing COMPASS forcefield, a part of BIOVIA Materials Studio package [51]. Adsorption Locator identifies possible configurations by carrying out Monte Carlo searches of the configurational space of the guest-host system as the temperature is slowly reduced according to a simulated annealing schedule. This process is repeated to identify further local energy minima. The lowest energy generated structures of each of the complexes and the structures of the substrates (caffeine, α-CD, β-CD, and γ-CD) were further optimized at the DFT level of theory.

DFT calculations were performed using the Gaussian 16 software [52]. All-electron calculations were done using 6-311++G(d,p) Gaussian basis sets, as the 6-31G family of basis sets is often used to analyze the host-guest docking systems as the ones in this study. B3LYP functional with the Grimme’s dispersion force corrections (B3LYP-D3) was used in our calculations as this method has been recently proven to provide accurate results for the studies of similar systems (complexes of plumbagin with CDs) [40]. The basis set superposition error (BSSE) was corrected with the counterpoise method. The polarizable continuum model (PCM) [53] was used to model solvation effects for water as the solvent (dielectric constant equals 78.540). The natural mode frequencies were calculated in harmonic approximation to confirm that each structure was not in a transition state. The existence of only positive frequencies confirmed the findings.

To investigate the binding energy changes (∆*E*_bind_) resulting from complexation between the guest (caffeine) and the host (CD) molecules, calculations were performed according to the equation:

∆*E*_bind_ = ∆*E*(complex)_opt_ − [∆*E*(host)_opt_ + ∆*E*(guest)_opt_],

where ∆*E*(complex)_opt_, ∆*E*(host)_opt_, and ∆*E*(guest)_opt_ represent the total optimized energy of the complex, the free host and the free guest, respectively. 

### 3.8. Toxicity

Fish Embryo Acute Toxicity (FET) test was done as described in the OECD guidelines, test no. 236 [54] with some modifications. In short, eggs were collected from naturally spawned crosses (1:1 female to male ratio) of TL and albino lines. Embryos from a minimum of 4 pairs were pulled out, washed thoroughly with E3 medium, transferred into a *ϕ* 9 cm Petri dish with fresh E3 medium and incubated at 28 °C. Eggs were visually inspected at blastula stage. Only batches with the overall fertilization rate ≥ 80% were used. At 4 hpf or 2 dpf, high-quality embryos were selected and manually transferred with a minimum volume of E3 to polystyrene 24-well plates (SARSTEDT) at a density of 1 egg/well in 1mL of liquid. The tested compounds were dissolved in E3 at concentrations of up to 5 mM. For each treatment, one plate with 24 eggs was used. The toxicities of all three Caf@CDs were tested. As controls, E3, Caf, and pure CDs were used. The experiment lasted for 1 day and during that time the embryos were kept in static conditions in darkness at 28 °C. After 20–24 h, developmental abnormalities and mortality were scored. The experiment was repeated 2–6 times with similar results. 

### 3.9. Caffeine Uptake

For the uptake study, groups of 50 (at 3 dpf) or 100 (at 4 hpf) embryos were placed into 100 µm cell stringers (Biologix) and transferred into a 9 cm Petri dish filled with 20–30 mL of E3 substituted with caffeine or Caf@CDs. To exclude the possibility that the tested compounds would be swallowed but not absorbed by the zebrafish body, two stages (4 hpf and 3 dpf) were chosen. At those stages, the mouth was not yet open. The 50 µM concentration of caffeine and Caf@CDs was chosen as the minimum tested concentration as it is near the minimum detection limit for HPLC. At different time-points from 0 min to 24 h the embryos were rinsed thoroughly with E3 and transferred to an Eppendorf tube. After removing excess liquid, the embryos were frozen and stored at 20 °C until further processing. Prior to HPLC analysis the samples were thawed on ice and homogenized with an insulin syringe in 1 mL of MQ water. Homogenates were supplemented with acetic acid and methanol to final concentrations of 2% and 5%, respectively, and the samples were clarified by centrifugation for 3 min at 14,000× *g*. Caffeine was partially purified from the supernatants by solid-phase extraction (SPE) on a Strata-X 33 µm Polymeric Reverse Phase column at 30 mg/1 mL (Phenomenex). Clarified samples were loaded on conditioned columns and then the columns were washed with 1 mL of 10% methanol in water. The caffeine-containing fraction was eluted with two 0.5 mL washes of 50% methanol in water. The samples were concentrated to dryness in a centrifuge evaporator and dissolved in 50 µL of 5% methanol in water. The caffeine content was quantified on an ACQUITY UPLC system with a PDA eλ detector (Waters) using an ACQUITY UPLC BEH C18 1.7 µm column 2.1 × 100 mm (Waters). A total of 37.5 µL of sample was loaded, and elution was performed at 0.25 mL/min with the following profile: 0–6 min, 10% methanol in water; 6–21 min, 10–30% gradient of methanol in water with the monitoring of absorption at 272 nm. The caffeine peak eluted after 12 min and was quantified by measuring the peak area. A calibration curve was obtained by linear regression (R^2^ = 0.9910) from two series of homogenates of untreated embryos spiked with 50–3000 ng of pure caffeine and processed as described above. Each calibration sample was analyzed in triplicate.

### 3.10. Ethical Statement

Zebrafish (*Danio rerio*) originated from the Zebrafish Core Facility of the International Institute of Molecular and Cell Biology in Warsaw, Poland (license no. PL14656251 from the District Veterinary Inspectorate in Warsaw; licenses no. 064 for breeder and 0051 for user, both issued by the Ministry of Science and Higher Education in Poland). The animals were housed, bred and used in accordance with the Directive 2010/63/EU on the protection of animals used for scientific purposes and Polish Act of January 15, 2015 on the fundamental ethical principles for the protection of animals that are used for scientific or educational purposes. As the experiments were performed on zebrafish embryos younger than 120 hpf, they did not require permit from either the ethics committee or the institutional review board. To ensure that research complies with the commonly accepted ‘3Rs’, the assays based on zebrafish embryos at early stages of development were chosen. In the case of treatment leading to 100% mortality, the experiments were limited to two independent biological replicas.

## 4. Conclusions

Standard methods for obtaining API complexes with CDs in solid form can be quite laborious and time consuming. For example, cogrinding of APIs with CDs is usually carried out in high-energy mills for one hour or longer [55,56,57,58]. On the basis of the results reported in this work, it should be possible to use low-energy cogrinding, which is desirable due to the simplicity and reduced production time. Using the cogrinding method presented in this work, a time of 30 min is sufficient to obtain caffeine complexes with α-CD and β-CD. Moreover, evaporation-induced deposition appears to yield caffeine complexes with γ-CD and is therefore a potential alternative to freeze-drying.

In the research reported here, multiple analytical methods (PXRD, DSC, FT-IR and Raman spectroscopy) were used, as well as theoretical methods (molecular modeling). The results of each method were described and discussed in detail. Eventually, the methods used to obtain the complexes were found to be effective.

In vivo studies showed increased toxicity of caffeine when complexed with CDs. This effect is primarily demonstrated as increased mortality of the treated zebrafish embryos. According to HPLC analysis, the enhanced toxicity of the complexes is not the result of an increased caffeine uptake when administered in complexed form. Synergistic toxicity could be a plausible explanation of the observed effects. However, further verification is needed to support it. It also remains to be seen whether Caf@CDs have similar effects on warm-blooded organisms.

Apart from further in-depth studies on the toxicity of Caf@CDs, it is especially worthwhile to implement the application of complexed caffeine. The benefits usually ascribed to complexation of APIs by CDs are still open to be addressed in this case. As we showed the successful synthesis of Caf@CDs, these solids can be examined towards, e.g., enhanced solubility and stability. Moreover, it would also be interesting to find whether or not bitter taste of caffeine was masked by complexing. If future studies confirm the enhanced bioactivity and reduced bitterness of caffeine when complexing with CDs, then new possibilities in the pharmaceutical and food industry will open up.

## Figures and Tables

**Table 1:** Shorthand notations for the substances under investigation.

Substance (Method of Preparation)		α-CD	β-CD	γ-CD
single component (ground, dry)		α-CD1	β-CD1	γ-CD1
single component (deposited from soln.)		α-CD3	β-CD3	γ-CD3
putative complex (coground, dry)		Caf@α-CD1	Caf@β-CD1	Caf@γ-CD1
putative complex (coground, w/water drops)		Caf@α-CD2	Caf@β-CD2	Caf@γ-CD2
putative complex (deposited from soln.)		Caf@α-CD3	Caf@β-CD3	Caf@γ-CD3
mixture (from dried CD)		Caf+α-CD1	Caf+β-CD1	Caf+γ-CD1
mixture (from deposited caffeine and CD)		Caf+α-CD3	Caf+β-CD3	Caf+γ-CD3
caffeine (as supplied)	Caf1	caffeine (deposited from soln.)		Caf3

**Table 2:** The initial, final, and maximum (i-f-m) temperatures (°C) of the DSC peaks, with the respective ∆*H* values (J⋅g^−1^, in parentheses).

Substance	Temperatures and Heats					Figures (Supplementary Material)
Caf1	148-169-159 (31)	233-248-239 (195)				S4, S9, and S14
Caf3	135-165-152 (24.5)	230-244-238 (193)				S6, S11, and S16
α-CD1	66-86-76 (16)	157-180-158 (8)	180-241-184 (270)			S4
Caf+α-CD1	57-70-63 (1.9)	133-174-152 (124)	234-242-236 (23)			S4 and S5
Caf@α-CD1	53-65-50 (0.5)	139-170-148 (11)	203-215-209 (11) *	230-241-234 (34)		S5 and S8
Caf@α-CD2	83-104-92 (30)	152-172-156 (36)	176-204-182 (143)	224-239-233 (28)		S8
α-CD3	78-99-89 (52)	138-140-143 (2.3)	174-222-176 (256)			S6
Caf+α-CD3	78-98-88 (38)	99-106-101 (2.2)	141-147-142 (2.4)	162-225-166 (267)	229-245-235 (28)	S6 and S7
Caf@α-CD3	78-94-87 (34)	145-150-146 (2.3)	154-184-160 (123)	184-209-199 (29)	223-237-231 (21)	S7 and S8
β-CD1	162-187-163 (255)					S9
Caf+β-CD1	68-150-123 (310)	104-150-123 (157)	154-168-161 (1.8)	235-241-237 (4.4)	268-280-270 (16)	S9 and S10
Caf@β-CD1	70-117-170 (265)	208-224-209 (13) *	225-243-232 (2.4) *	250-279-265 (22)		S10 and S13
Caf@β-CD2	145-161-148 (44)	178-196-175 (338)	230-238-233 (9)			S13
β-CD3	142-151-144 (7.5)	175-220-192 (493)				S11
Caf+β-CD3	141-188-143 (476)	233-240-236 (8.6)	257-271-265 (7)			S11 and S12
Caf@β-CD3	147-171-153 (35)	180-202-182 (250)	232-239-235 (4)			S12 and S13
γ-CD1	40-53-47 (1.7)	184-189-185 (2.2)	221-251-225 (80)			S14
Caf+γ-CD1	150-161-152 (5)	161-181-164 (89)				S14 and S15
Caf@γ-CD1	163-174-164 (12)	205-221-207(106)				S15 and S18
Caf@γ-CD2	178-208-183 (178)					S18
γ-CD3	104-115-108 (4.1)	132-162-147 (23.5)	174-217-177 (413)			S16
Caf+γ-CD3	96-110-105 (9.4)	119-131-126 (7)	148-157-149 (11)	172-194-173 (290)		S16 and S17
Caf@γ-CD3	168-199-172 (150)					S17 and S18

* exothermic events.

**Table 3:** Complexation energies of Caf@CDs calculated using the B3LYP-D3 DFT method, including the PCM water solvation scheme.

	∆*E*_bind_ (kcal-mol^−1^)	∆*E*_bind_/PCM (kcal-mol^−1^)
Caf@α-CD	−12.82	−8.56
Caf@β-CD	−25.88	−20.04
Caf@γ-CD	−30.08	−34.65

## Supplementary Materials

The following are available online at https://www.mdpi.com/article/10.3390/ijms22084191/s1: copies of the PXRD patterns, DSC thermograms (and their descriptions), and FT-IR and Raman spectra.
