# Supplementary material for: Caffeine-Cyclodextrin Complexes as Solids: Synthesis, Biological and Physicochemical Characterization
Source: Int J Mol Sci. 2021 Apr 18;22(8):4191. doi: 10.3390/ijms22084191 (PMC8073077; doi:10.3390/ijms22084191)
Supplement: Supplementary file 1 [file ijms-22-04191-s001.zip › ijms-1148924-supplementary.pdf]

Supporting material

for

Caffeine-cyclodextrin complexes as solids: Synthesis, biological and physicochemical characterization

Sebastian Szmeja [1], Tomasz Gubica [1,*], Andrzej Ostrowski [2], Aldona Zalewska [2], Łukasz Szeleszczuk [1], Katarzyna Zawada [1], Monika Zielińska-Pisklak [3], Krzysztof Skowronek [4] and Małgorzata Wiweger [4]

[1] *Department of Physical Chemistry, Chair of Physical Pharmacy and Bioanalysis, Faculty of Pharmacy, Medical University of Warsaw, Banacha 1, 02-097 Warsaw, Poland*
[2] *Faculty of Chemistry, Warsaw University of Technology, Noakowskiego 3, 00-664 Warsaw, Poland*
[3] *Department of Biomaterials Chemistry, Chair and Department of Inorganic and Analytical Chemistry, Faculty of Pharmacy, Medical University of Warsaw, Banacha 1, 02-097 Warsaw, Poland*
[4] *International Institute of Molecular and Cell Biology in Warsaw, Księcia Trojdena 4, 02-109 Warsaw, Poland*

*E-mail: tomasz.gubica@wum.edu.pl

Table of contents

Figure	Caption	p.
**S1**	PXRD patterns for Caf@α-CD, Caf+α-CD, and their components: (a) method 1; (b) method 3.	S3
**S2**	PXRD patterns for Caf@β-CD, Caf+β-CD, and their components: (a) method 1; (b) method 3.	S4
**S3**	PXRD patterns for Caf@γ-CD, Caf+γ-CD, and their components: (a) method 1; (b) method 3.	S5
**S4**	DSC thermograms for Caf1, α-CD1, and Caf+α-CD1.	S6
**S5**	DSC thermograms for Caf+α-CD1 and Caf@α-CD1.	S6
**S6**	DSC thermograms for Caf3, α-CD3, and Caf+α-CD3.	S7
**S7**	DSC thermograms for Caf+α-CD3 and Caf@α-CD3.	S7
**S8**	DSC thermograms for Caf@α-CD1, Caf@α-CD2, and Caf@α-CD3.	S8
**S9**	DSC thermograms for Caf1, β-CD1, and Caf+β-CD1.	S8
**S10**	DSC thermograms for Caf+β-CD1 and Caf@β-CD1.	S9
**S11**	DSC thermograms for Caf3, β-CD3, and Caf+β-CD3.	S9
**S12**	DSC thermograms for Caf+β-CD3 and Caf@β-CD3.	S10
**S13**	DSC thermograms for Caf@β-CD1, Caf@β-CD2, and Caf@β-CD3.	S10
**S14**	DSC thermograms for Caf1, γ-CD1, and Caf+γ-CD1.	S11
**S15**	DSC thermograms for Caf+γ-CD1 and Caf@γ-CD1.	S11
**S16**	DSC thermograms for Caf3, γ-CD3, and Caf+γ-CD3.	S12
**S17**	DSC thermograms for Caf+γ-CD3 and Caf@γ-CD3.	S12
**S18**	DSC thermograms for Caf@γ-CD1, Caf@γ-CD2, and Caf@γ-CD3.	S13
**S19**	FT-IR spectra for Caf@α-CD1, Caf+α-CD1, Caf1, and α-CD1.	S14

**S20**	FT-IR spectra for Caf@α-CD3, Caf+α-CD3, Caf3, and α-CD3.	S14
**S21**	FT-IR spectra for Caf@β-CD1, Caf+β-CD1, Caf1, and β-CD1.	S15
**S22**	FT-IR spectra for Caf@β-CD3, Caf+β-CD3, Caf3, and β-CD3.	S15
**S23**	FT-IR spectra for Caf@γ-CD1, Caf+γ-CD1, Caf1, and γ-CD1.	S16
**S24**	FT-IR spectra for Caf@γ-CD3, Caf+γ-CD3, Caf3, and γ-CD3.	S16
**S25**	Raman spectra for Caf@α-CD1, Caf+α-CD1, Caf1, and α-CD1.	S17
**S26**	Raman spectra for Caf@α-CD3, Caf+α-CD3, Caf3, and α-CD3.	S17
**S27**	Raman spectra for Caf@β-CD1, Caf+β-CD1, Caf1, and β-CD1.	S18
**S28**	Raman spectra for Caf@β-CD3, Caf+β-CD3, Caf3, and β-CD3.	S18
**S29**	Raman spectra for Caf@γ-CD1, Caf+γ-CD1, Caf1, and γ-CD1.	S19
**S30**	Raman spectra for Caf@γ-CD3, Caf+γ-CD3, Caf3, and γ-CD3.	S19
**S31**	Developmental defects caused by caffeine (Caf). (a) Representative images of PFA-fixed zebrafish embryos. Live embryos were exposed to caffeine, γ-CD or Caf@γ-CD from 36 till 48 hpf. The most severe morphological abnormalities can be noted in embryos exposed to caffeine. Caf@γ-CD had intermediate effect, whereas γ-CD had no effect on embryo development. Lateral view; scale bar, 1 mm; (b) measurements of body length of zebrafish embryos as shown in (a). Statistical significance between two groups was evaluated by the two-tailed unpaired *t*-test using the GraphPad Prism version 5.0 for Windows. Data are presented as mean values ± SD, n > 29 in each group.	S24
**S32**	Examples of the caffeine uptake by zebrafish embryos treated from 4 hpf with 50 μM caffeine, Caf@α-CD, Caf@β-CD (a) or Caf@γ-CD (a and b).	S25

Results of DSC measurements pp S20-S23

References p. S25

(a)

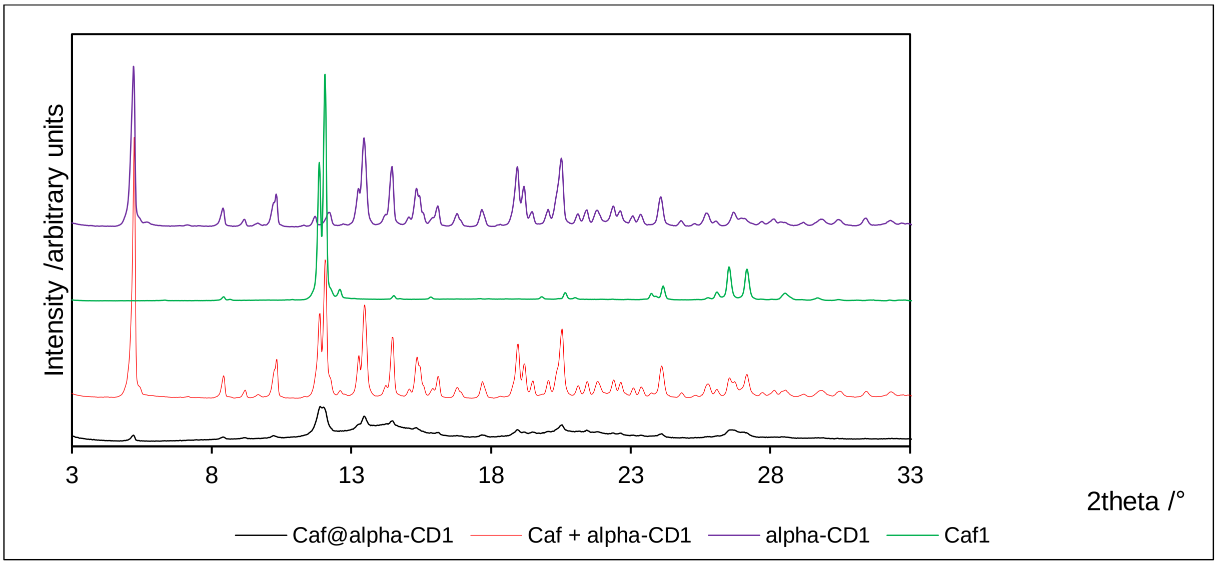

(b)

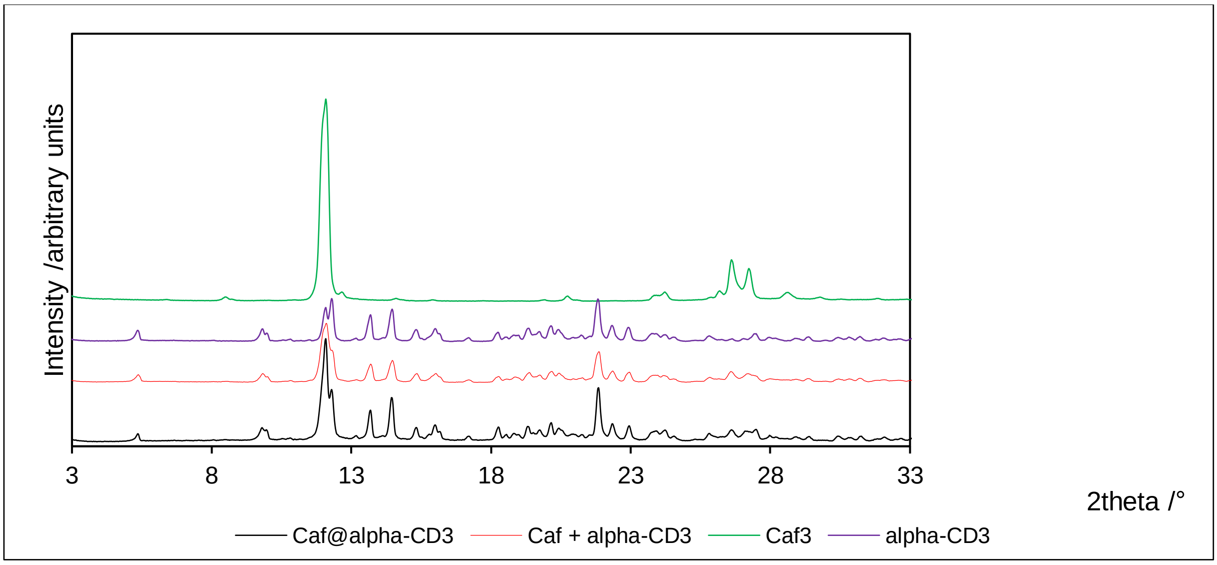

Figure S1. PXRD patterns for Caf@α-CD, Caf+α-CD, and their components: (a) method 1; (b) method 3.

(a)

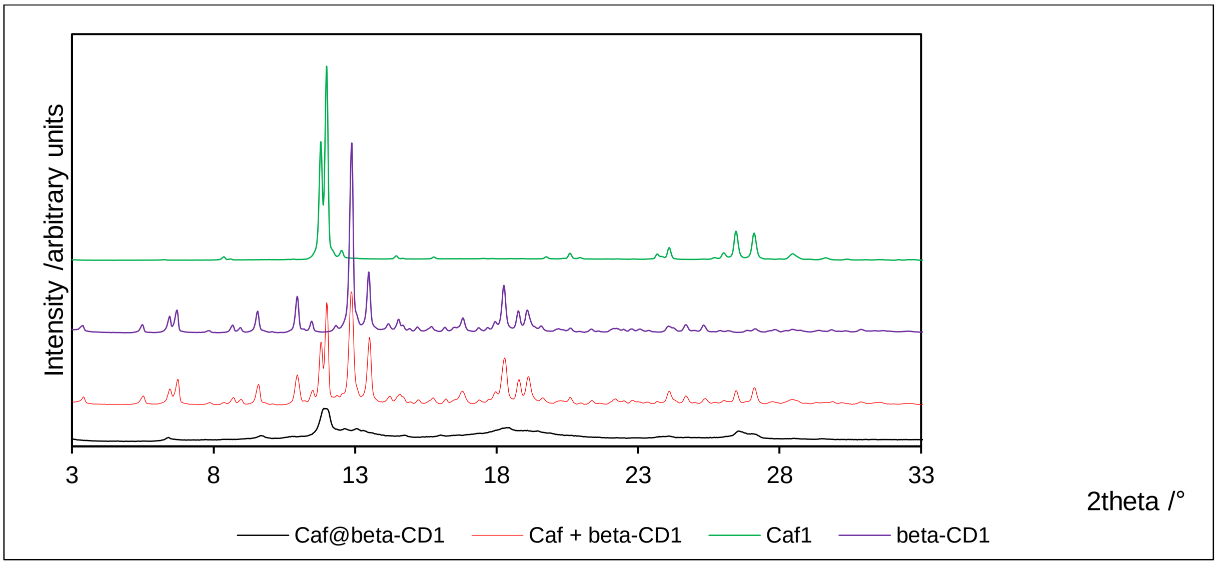

(b)

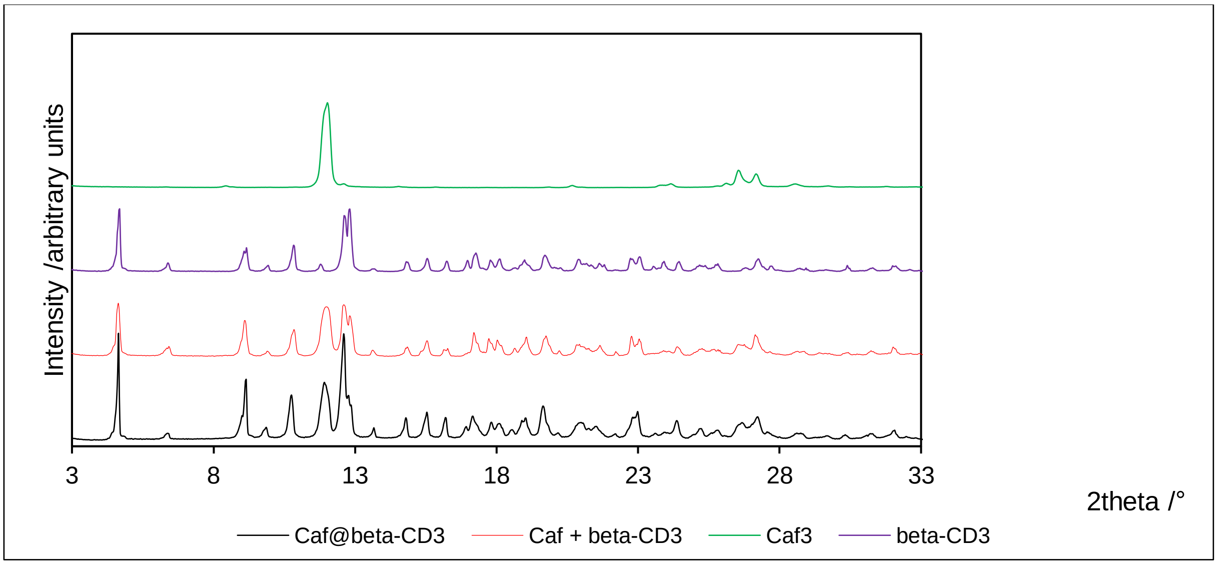

Figure S2. PXRD patterns for Caf@β-CD, Caf+β-CD, and their components: (a) method 1; (b) method 3.

(a)

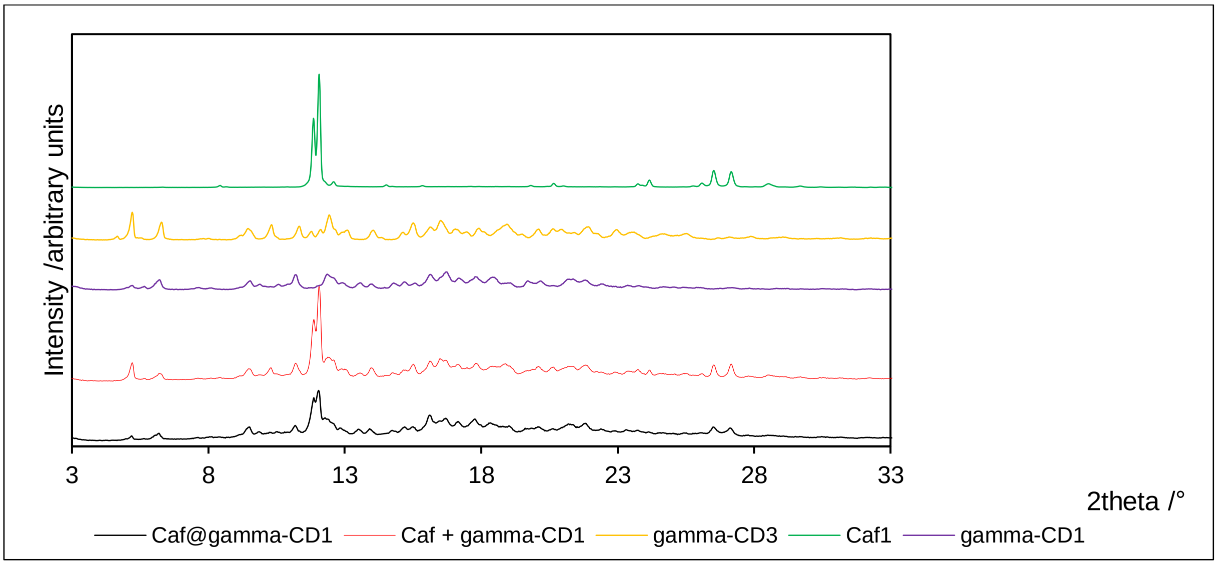

(b)

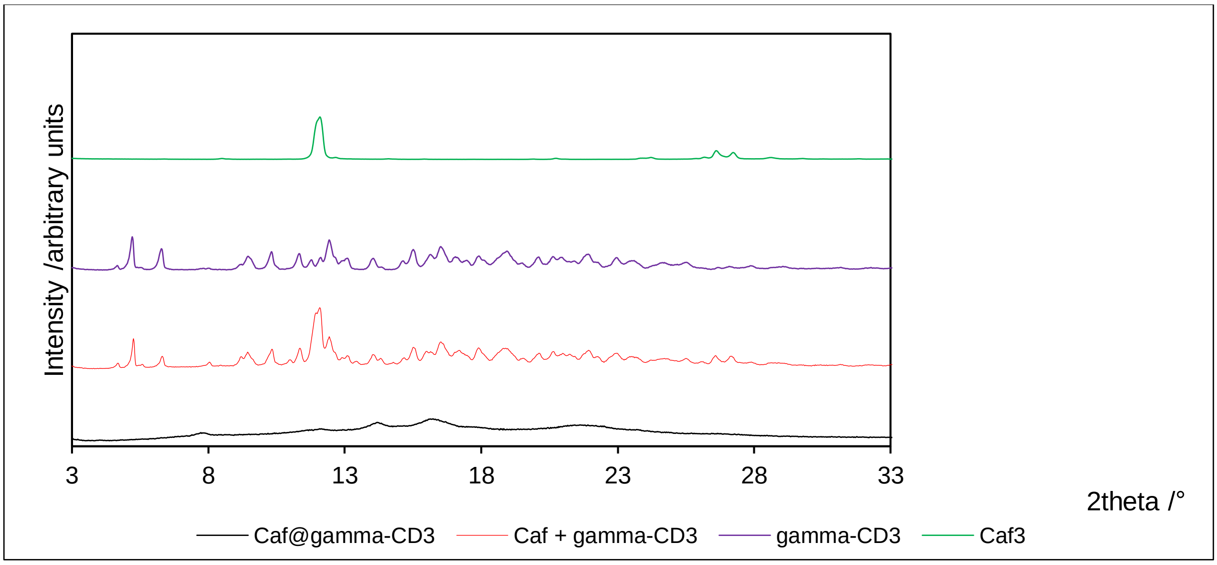

Figure S3. PXRD patterns for Caf@γ-CD, Caf+γ-CD, and their components: (a) method 1; (b) method 3.

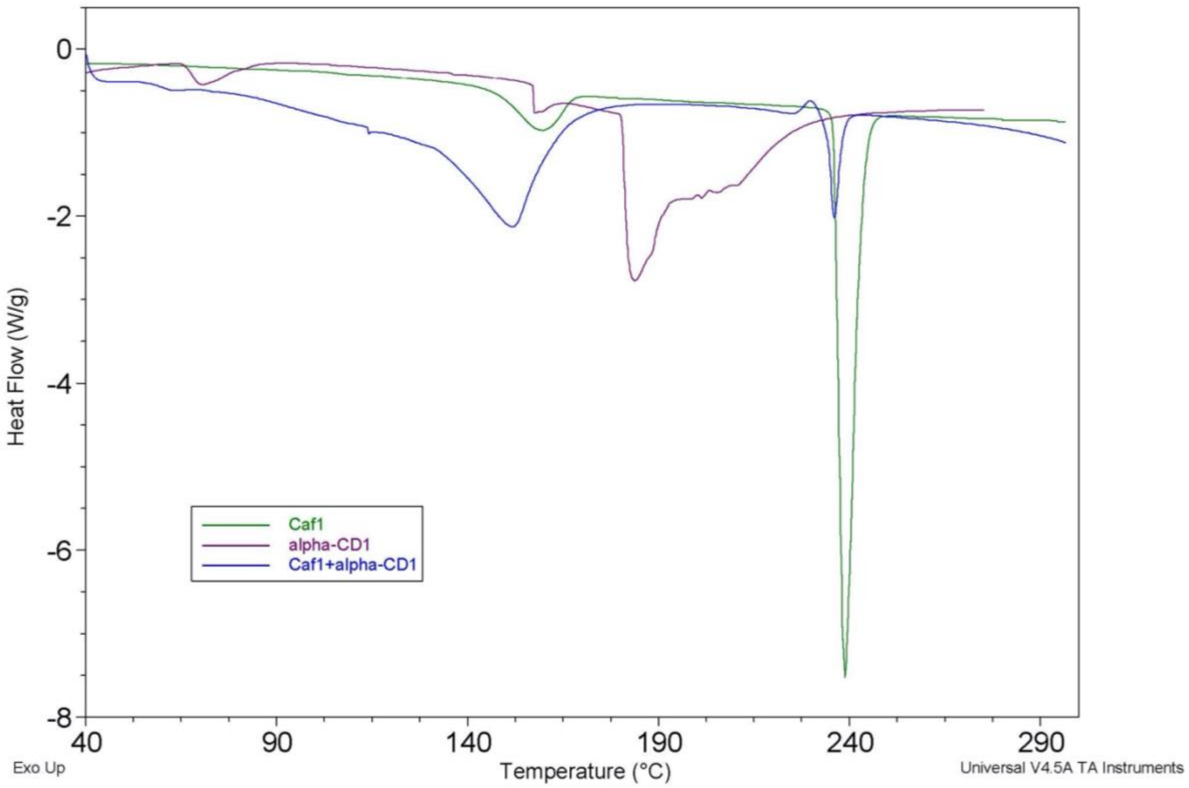

Figure S4. DSC thermograms for Caf1, α-CD1, and Caf+α-CD1.

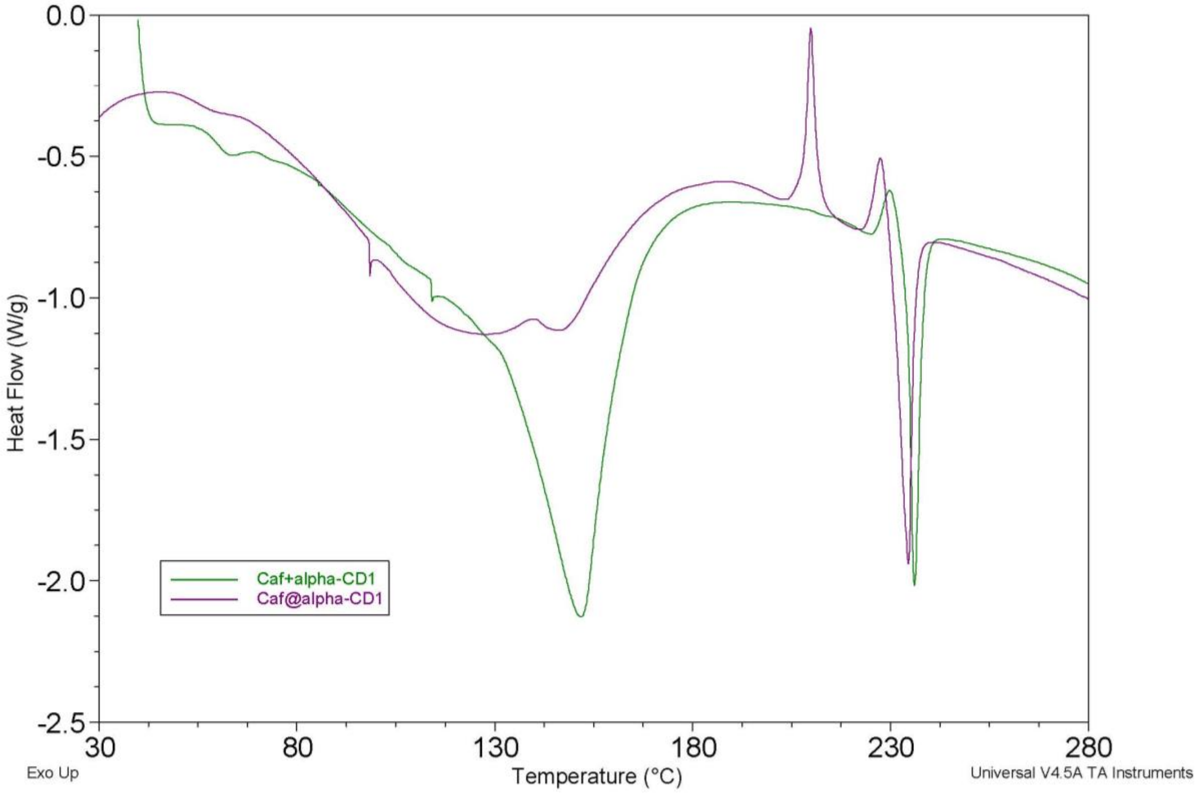

Figure S5. DSC thermograms for Caf+α-CD1 and Caf@α-CD1.

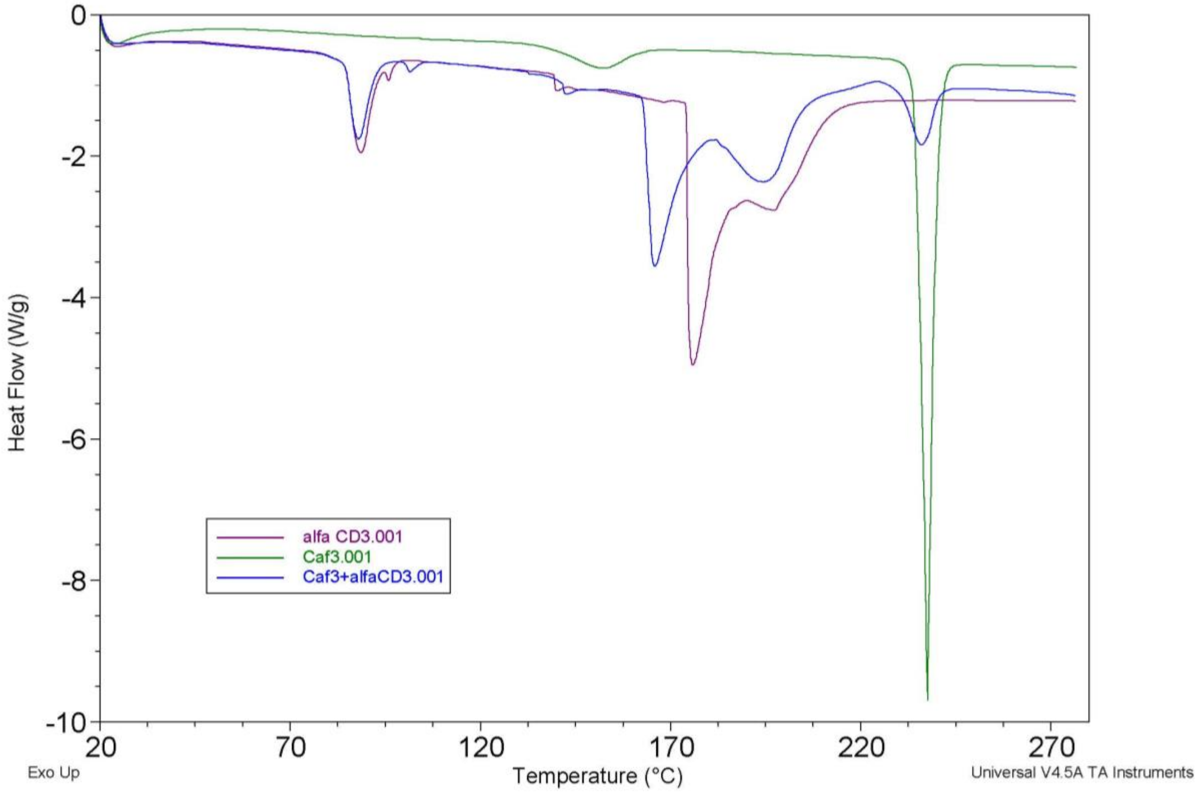

Figure S6. DSC thermograms for Caf3, α-CD3, and Caf+α-CD3.

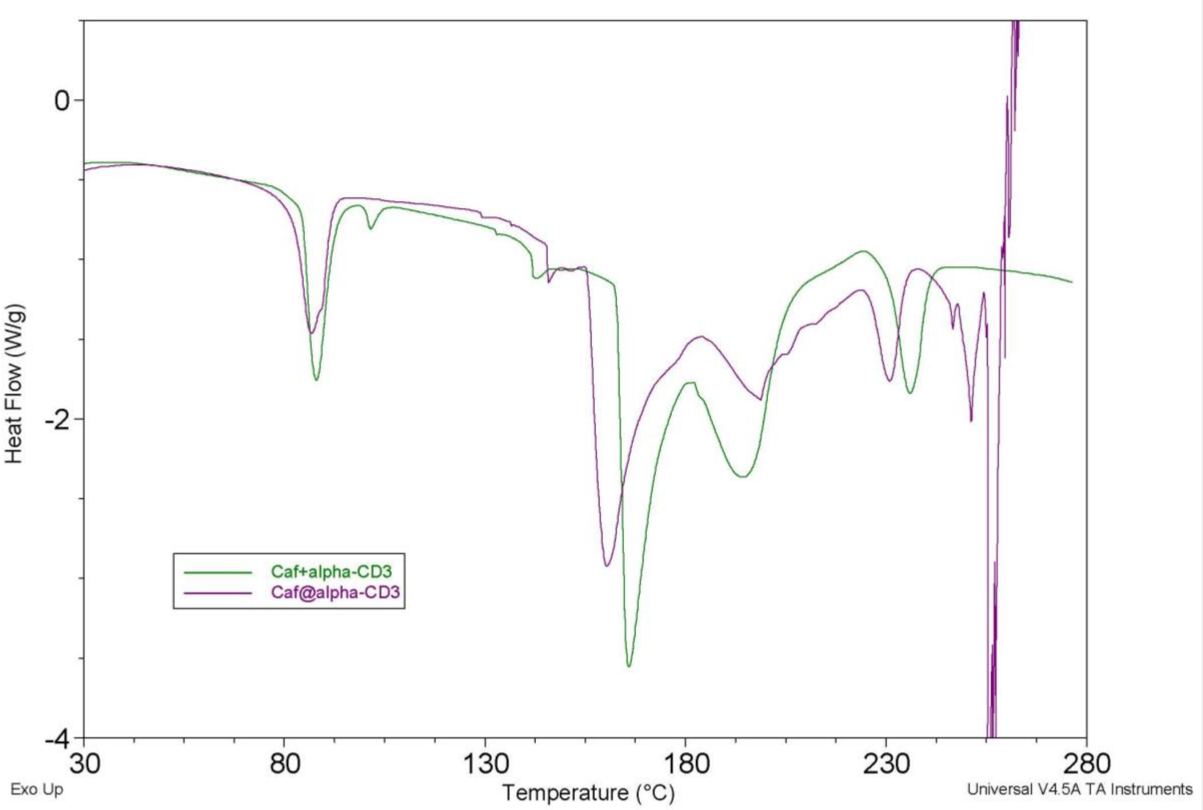

Figure S7. DSC thermograms for Caf+α-CD3 and Caf@α-CD3.

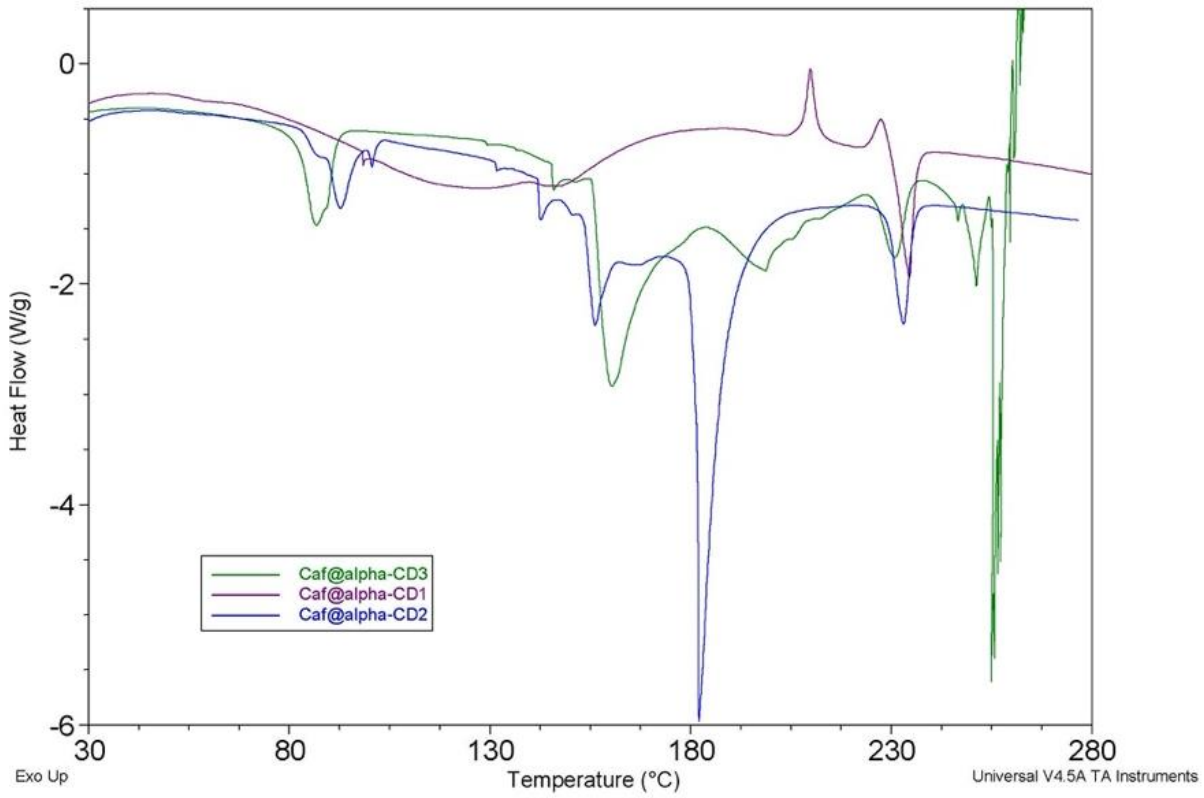

Figure S8. DSC thermograms for Caf@α-CD1, Caf@α-CD2, and Caf@α-CD3.

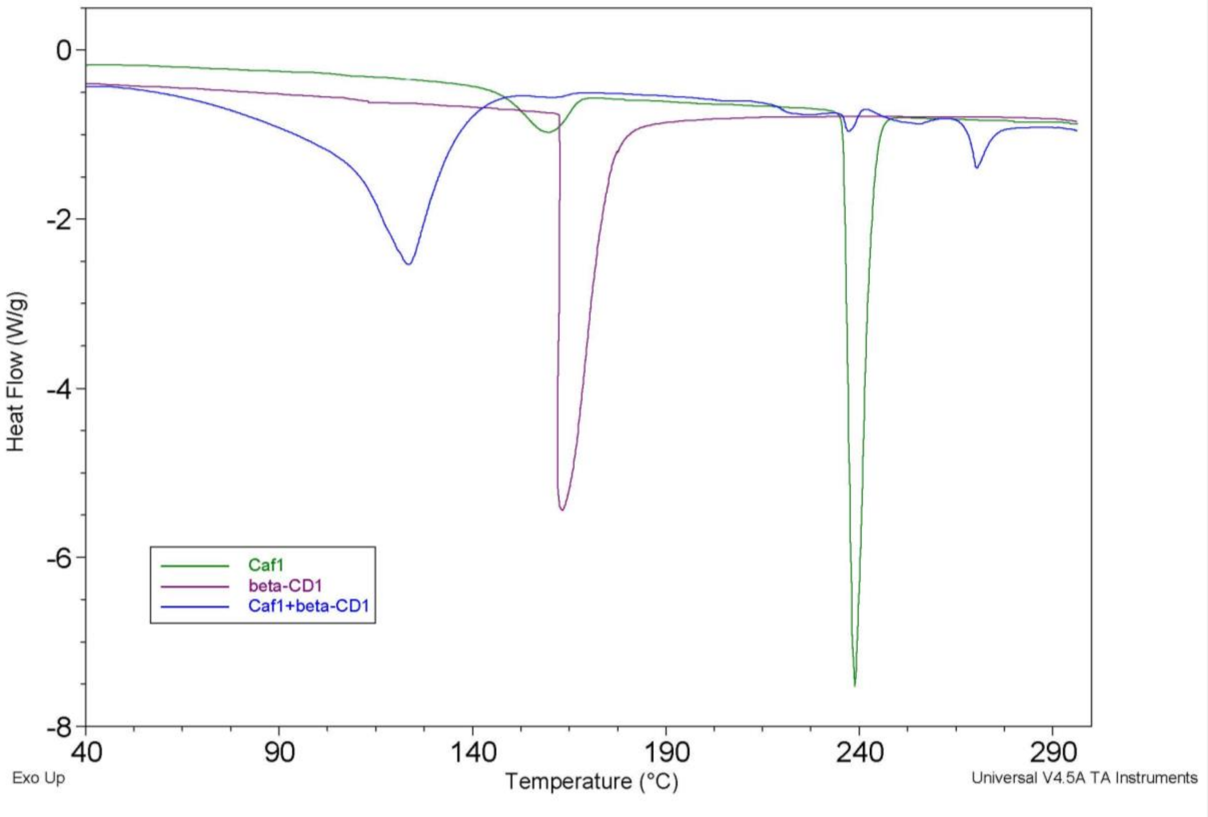

Figure S9. DSC thermograms for Caf1, β-CD1, and Caf+β-CD1.

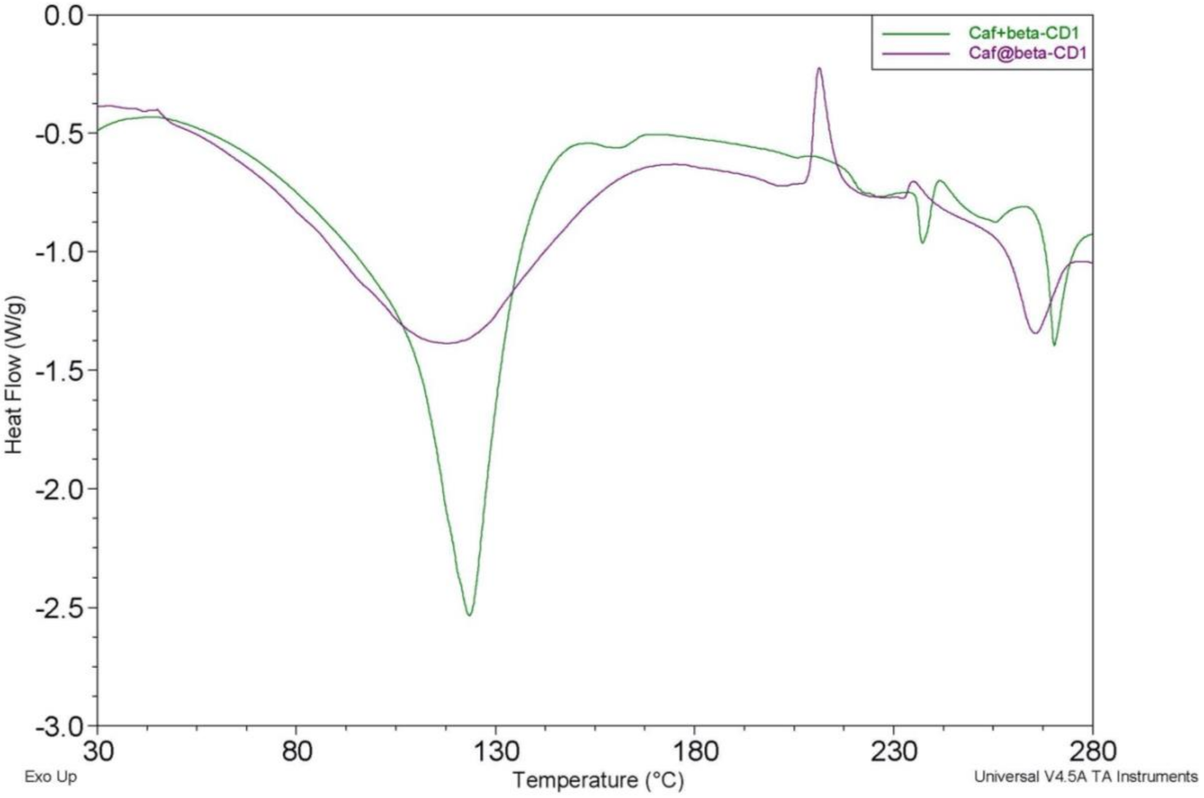

Figure S10. DSC thermograms for Caf+β-CD1 and Caf@β-CD1.

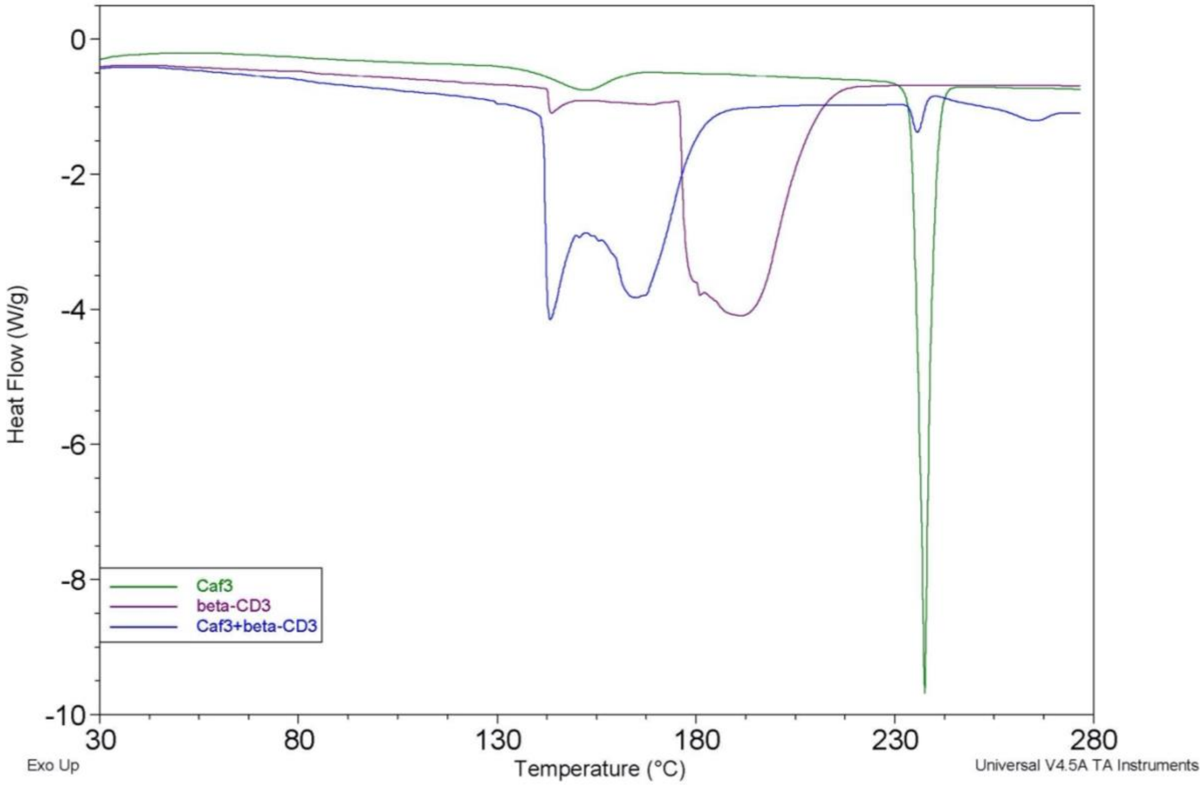

Figure S11. DSC thermograms for Caf3, β-CD3, and Caf+β-CD3.

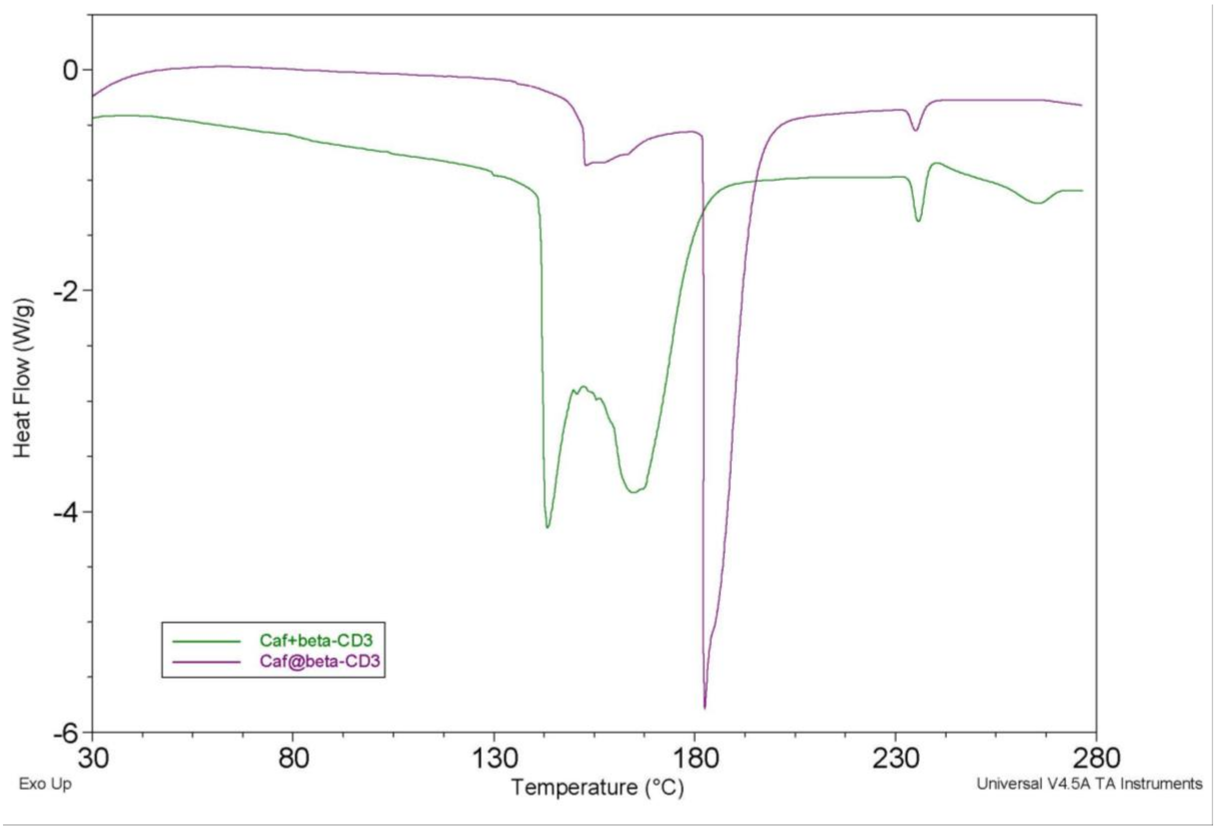

Figure S12. DSC thermograms for Caf+β-CD3 and Caf@β-CD3.

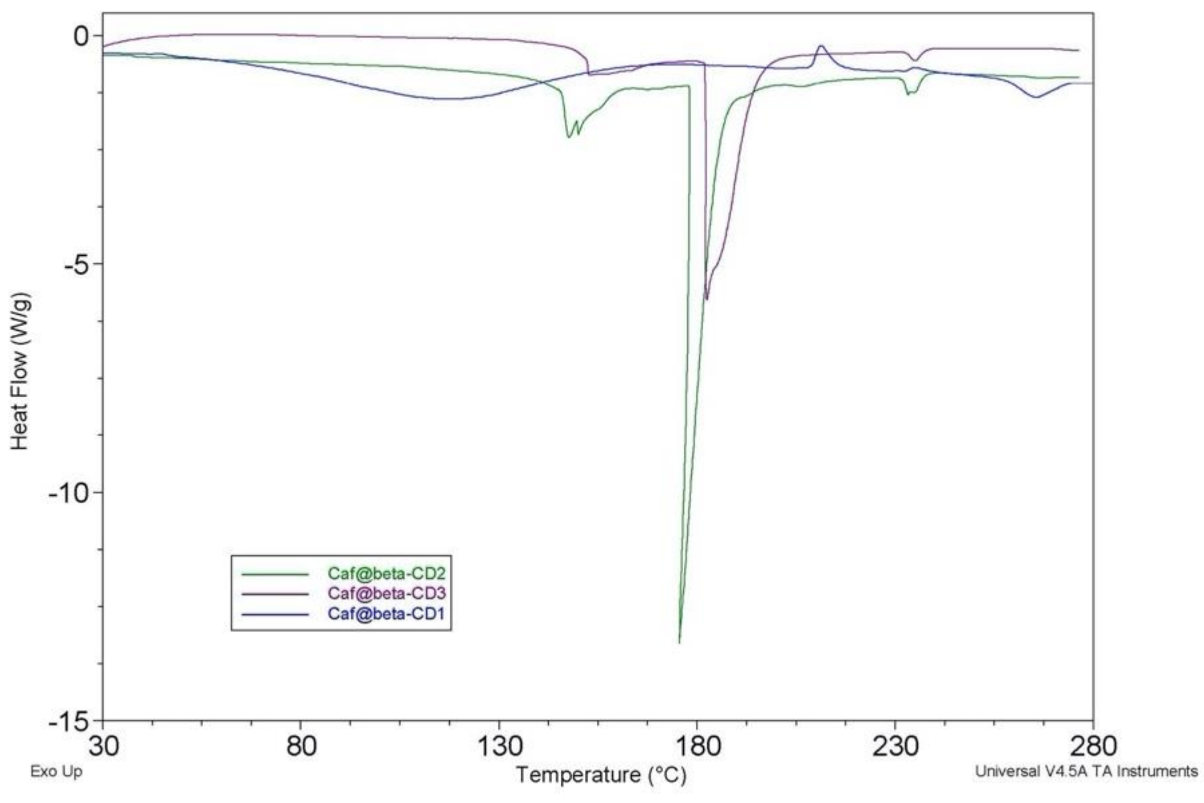

Figure S13. DSC thermograms for Caf@β-CD1, Caf@β-CD2, and Caf@β-CD3.

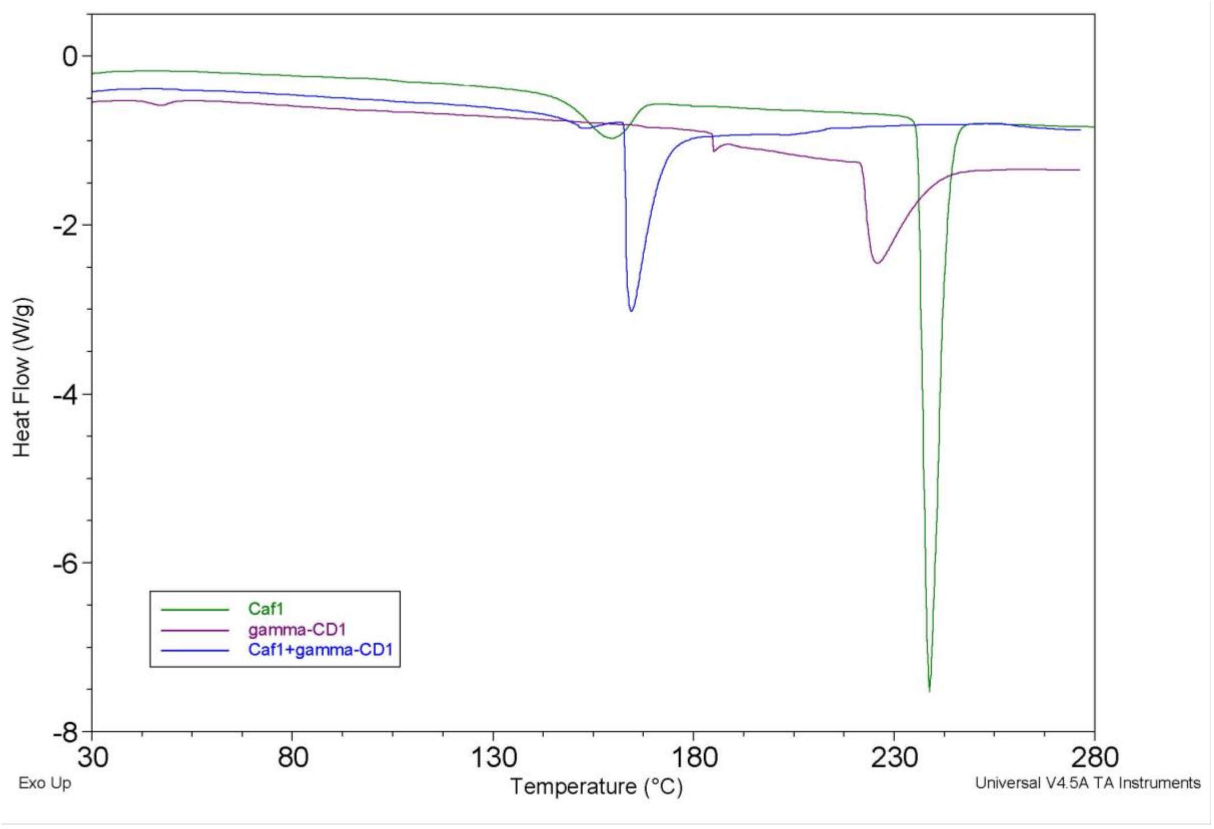

Figure S14. DSC thermograms for Caf1, γ-CD1, and Caf+γ-CD1.

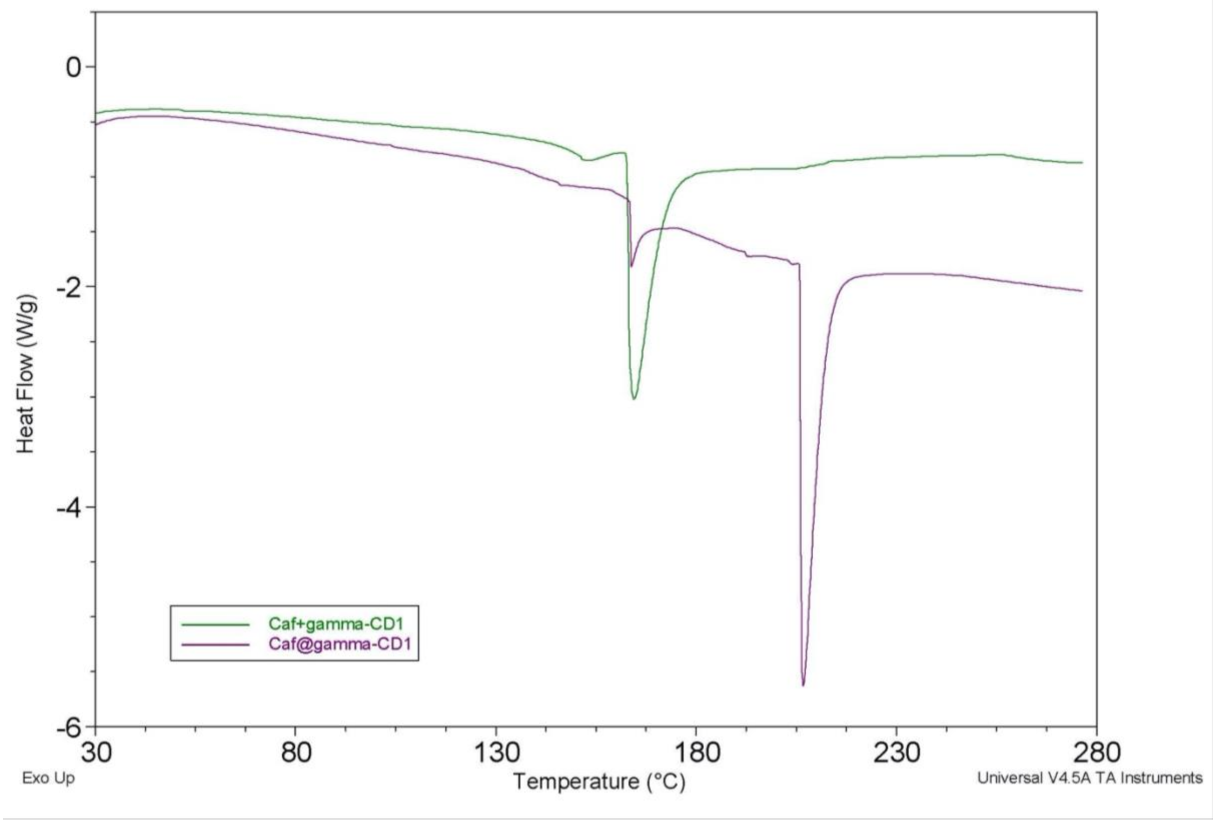

Figure S15. DSC thermograms for Caf+γ-CD1 and Caf@γ-CD1.

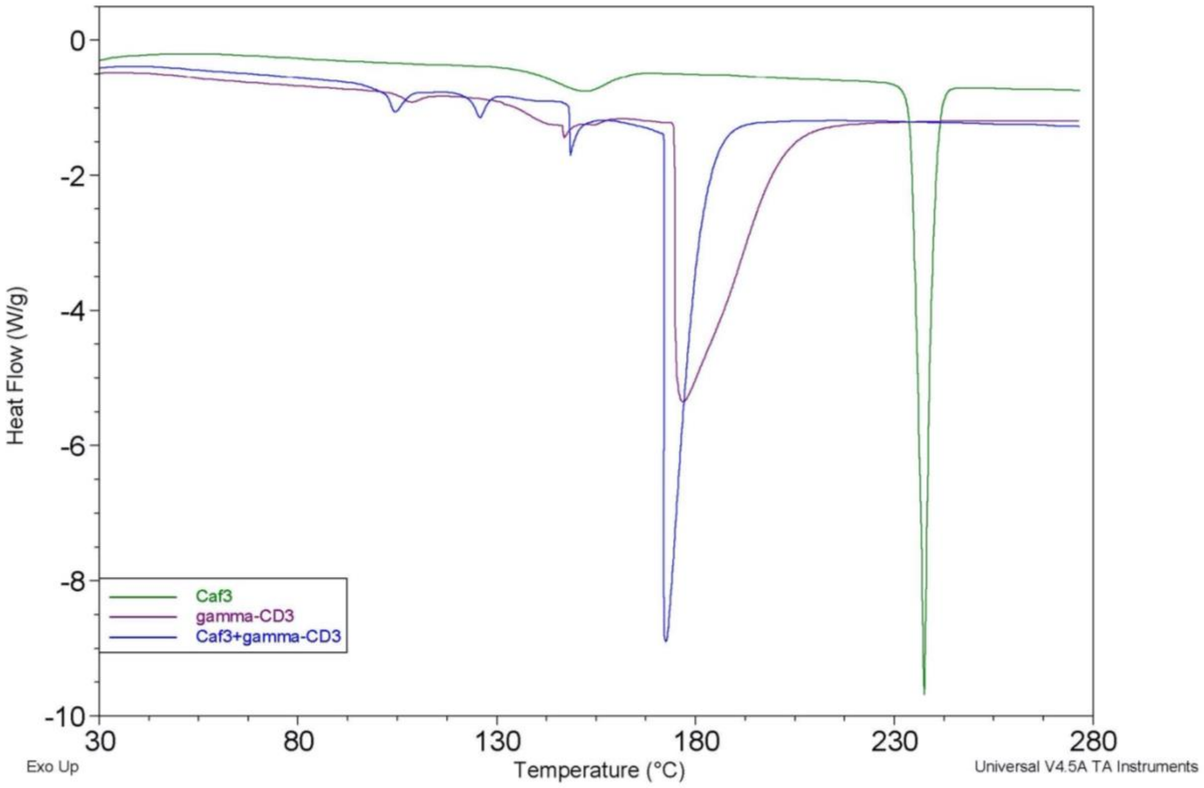

Figure S16. DSC thermograms for Caf3, γ-CD3, and Caf+γ-CD3.

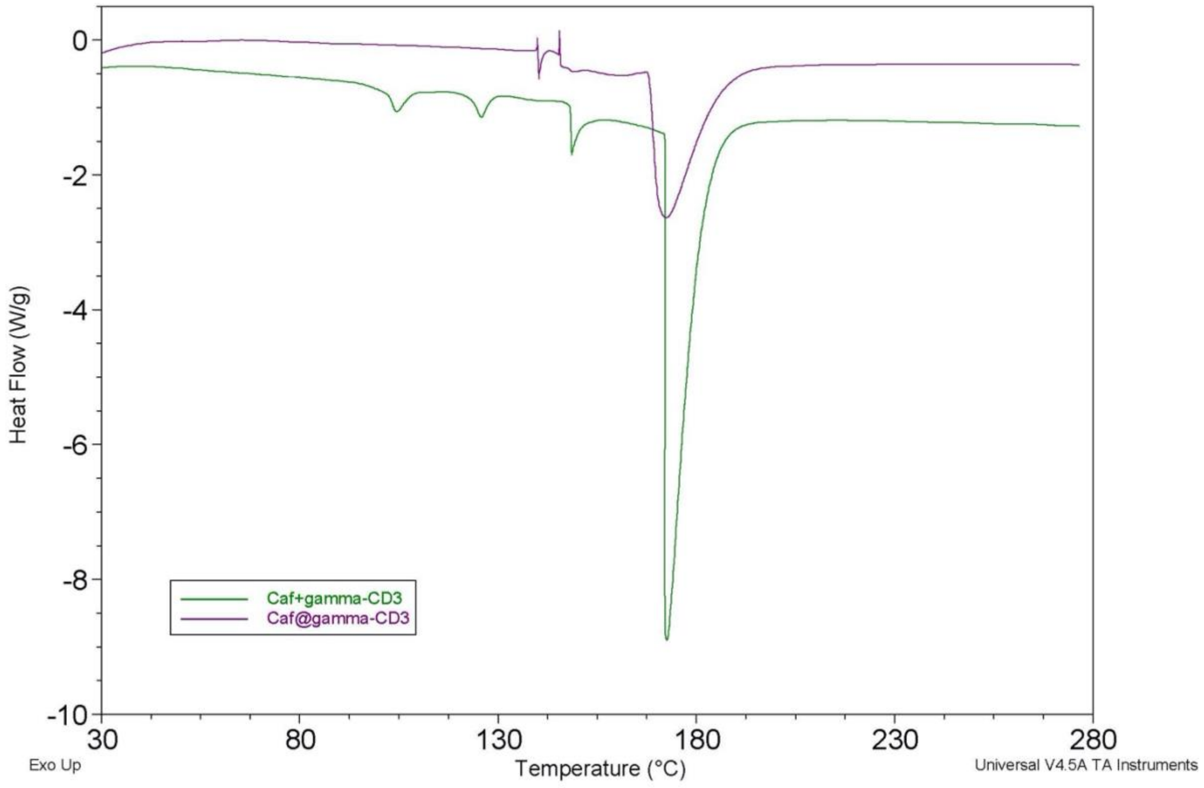

Figure S17. DSC thermograms for Caf+ γ-CD3 and Caf@γ-CD3.

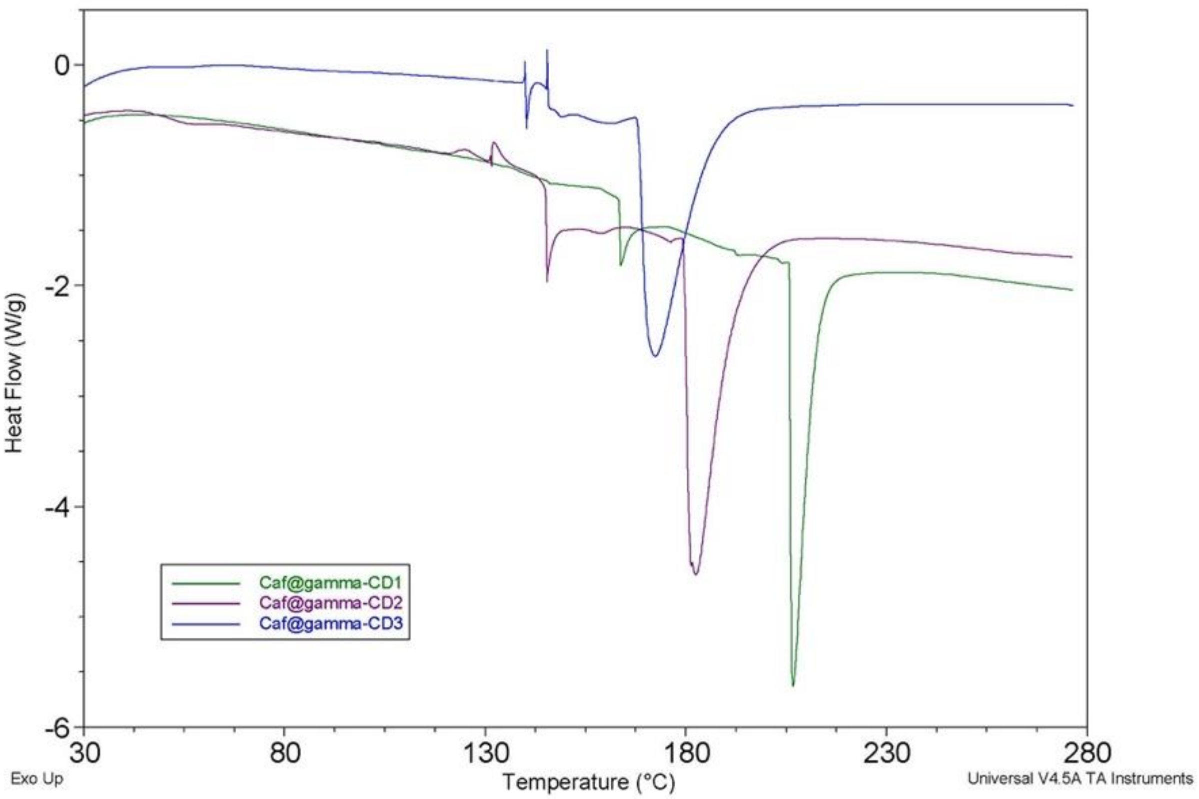

Figure S18. DSC thermograms for Caf@γ-CD1, Caf@γ-CD2, and Caf@γ-CD3.

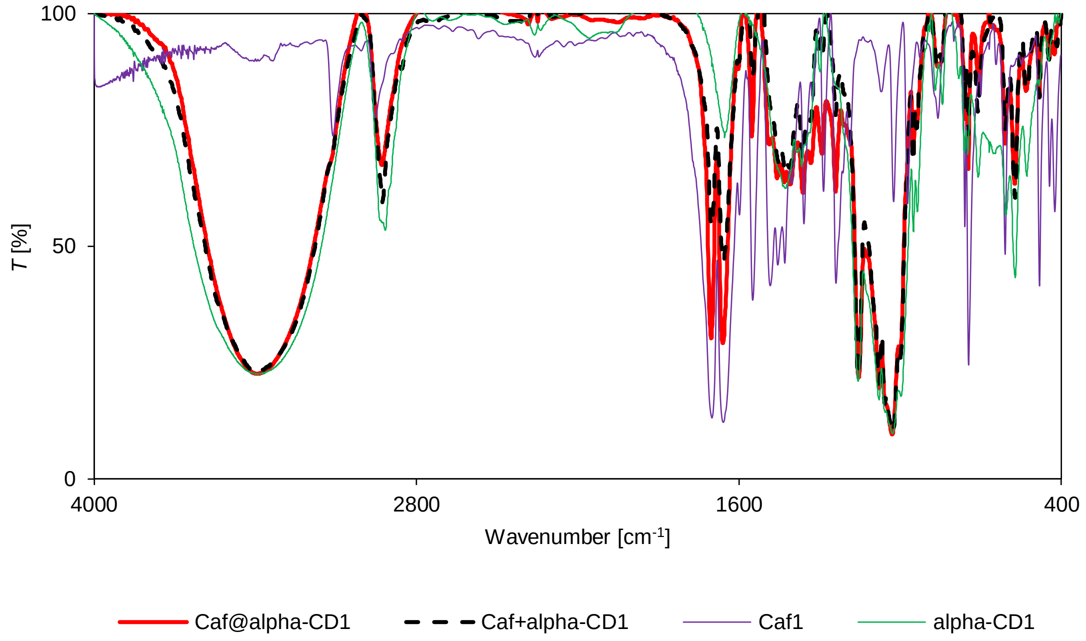

Figure S19. FT-IR spectra for Caf@α-CD1, Caf+α-CD1, Caf1, and α-CD1.

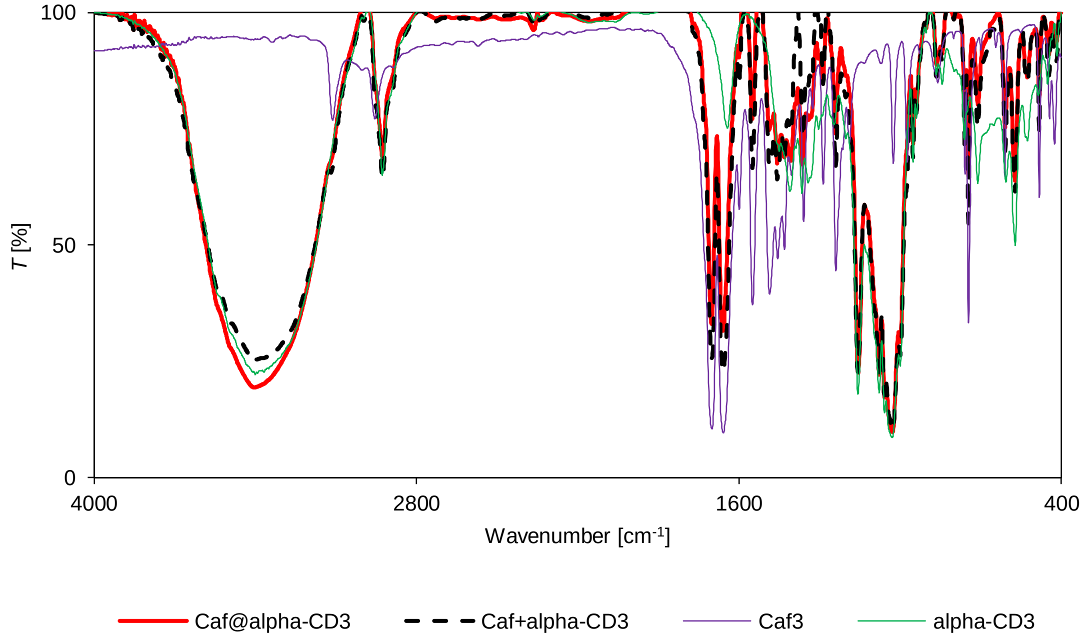

Figure S20. FT-IR spectra for Caf@α-CD3, Caf+α-CD3, Caf3, and α-CD3.

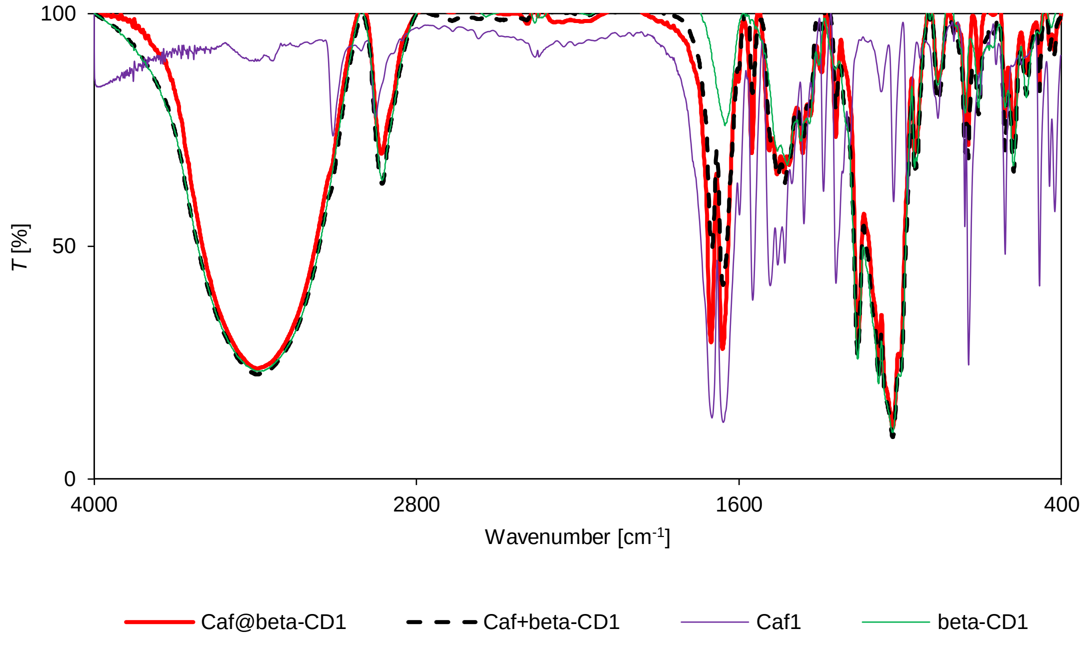

Figure S21. FT-IR spectra for Caf@β-CD1, Caf+β-CD1, Caf1, and β-CD1.

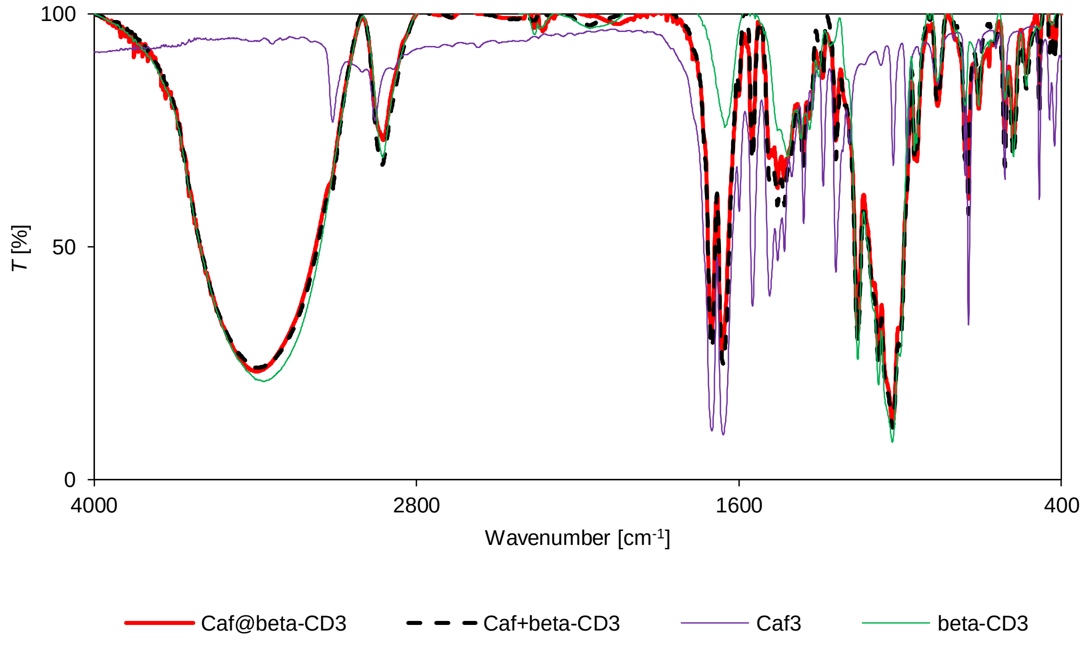

Figure S22. FT-IR spectra for Caf@β-CD3, Caf+β-CD3, Caf3, and β-CD3.

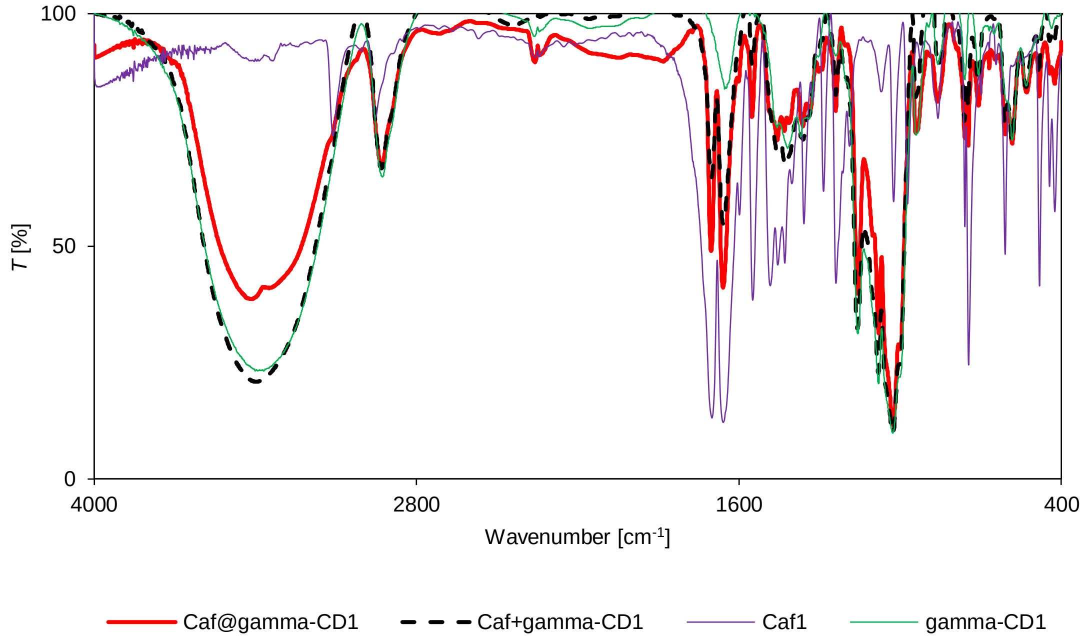

Figure S23. FT-IR spectra for Caf@γ-CD1, Caf+γ-CD1, Caf1, and γ-CD1.

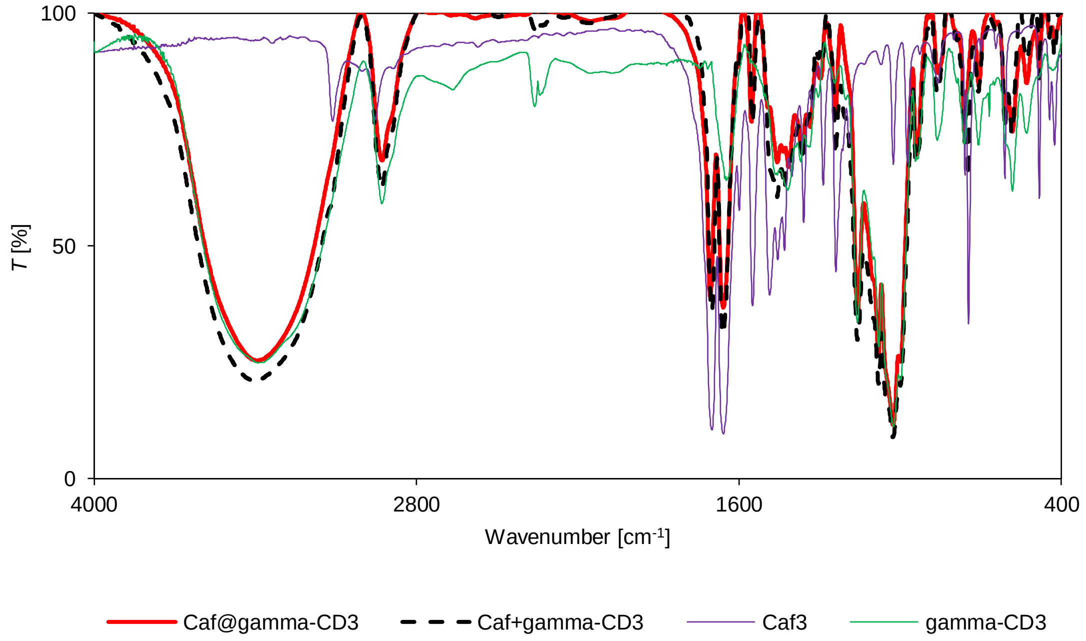

Figure S24. FT-IR spectra for Caf@γ-CD3, Caf+γ-CD3, Caf3, and γ-CD3.

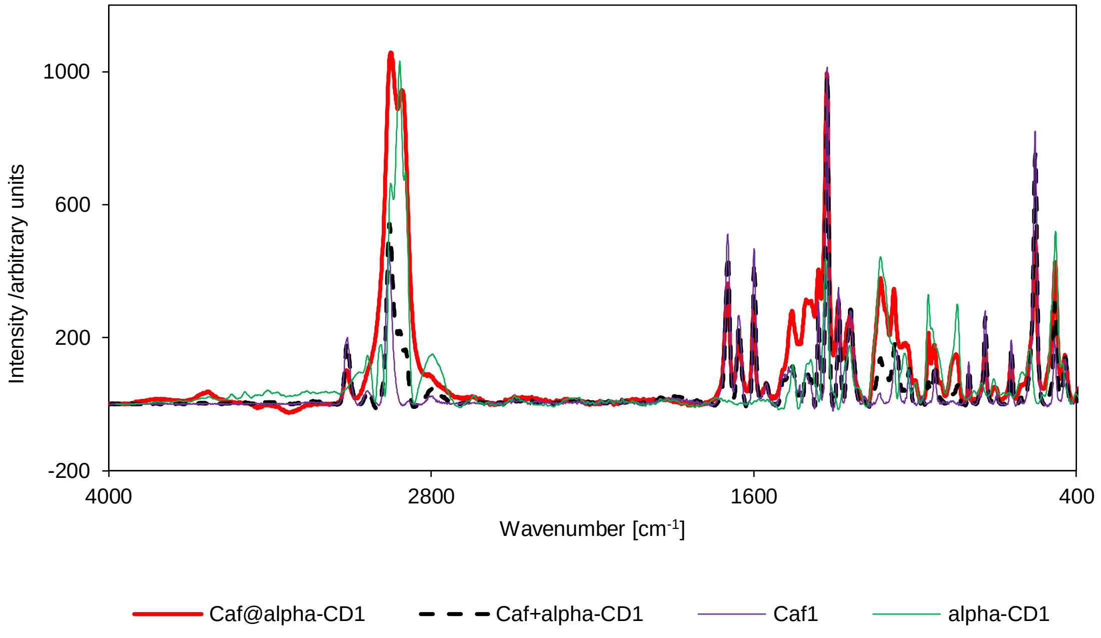

Figure S25. Raman spectra for Caf@α-CD1, Caf+α-CD1, Caf1, and α-CD1.

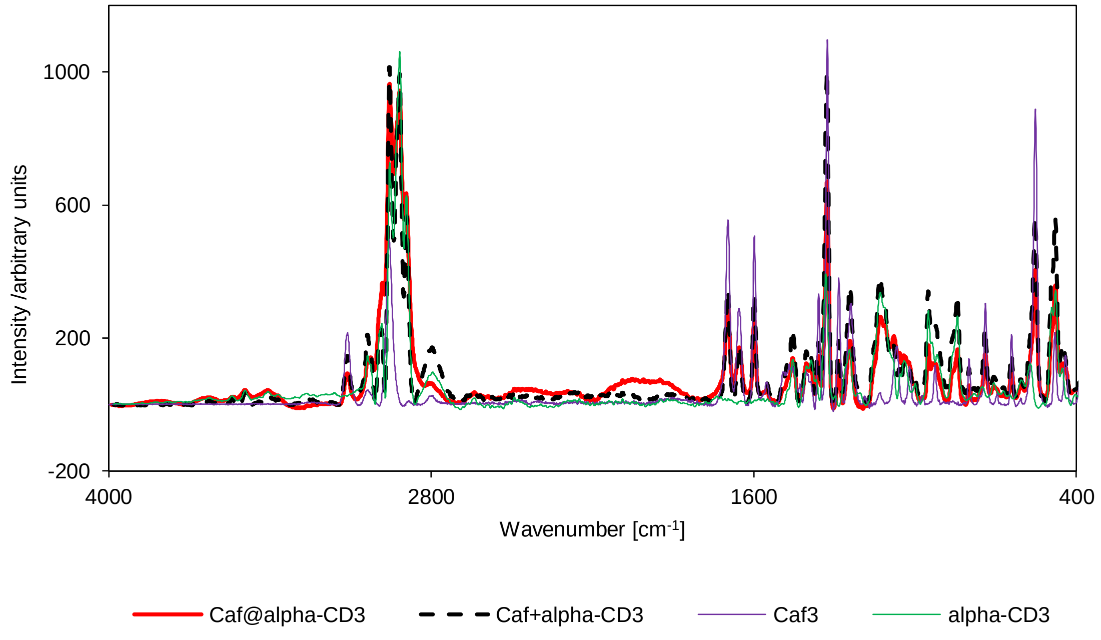

Figure S26. FT-IR spectra for Caf@α-CD3, Caf+α-CD3, Caf3, and α-CD3.

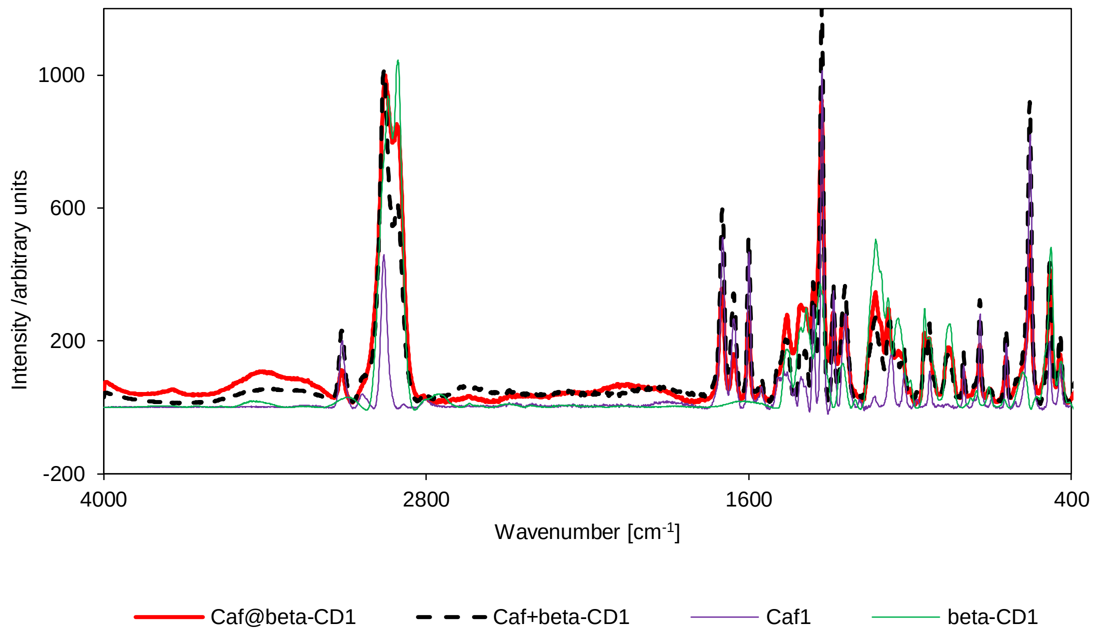

Figure S27. Raman spectra for Caf@β-CD1, Caf+β-CD1, Caf1, and β-CD1.

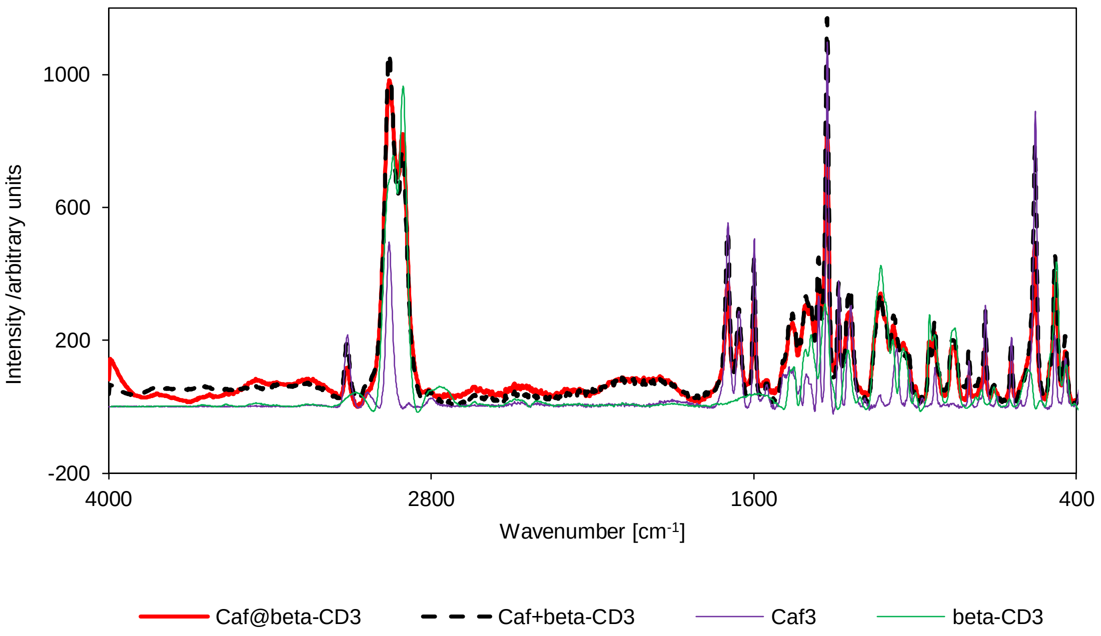

Figure S28. Raman spectra for Caf@β-CD3, Caf+β-CD3, Caf3, and β-CD3.

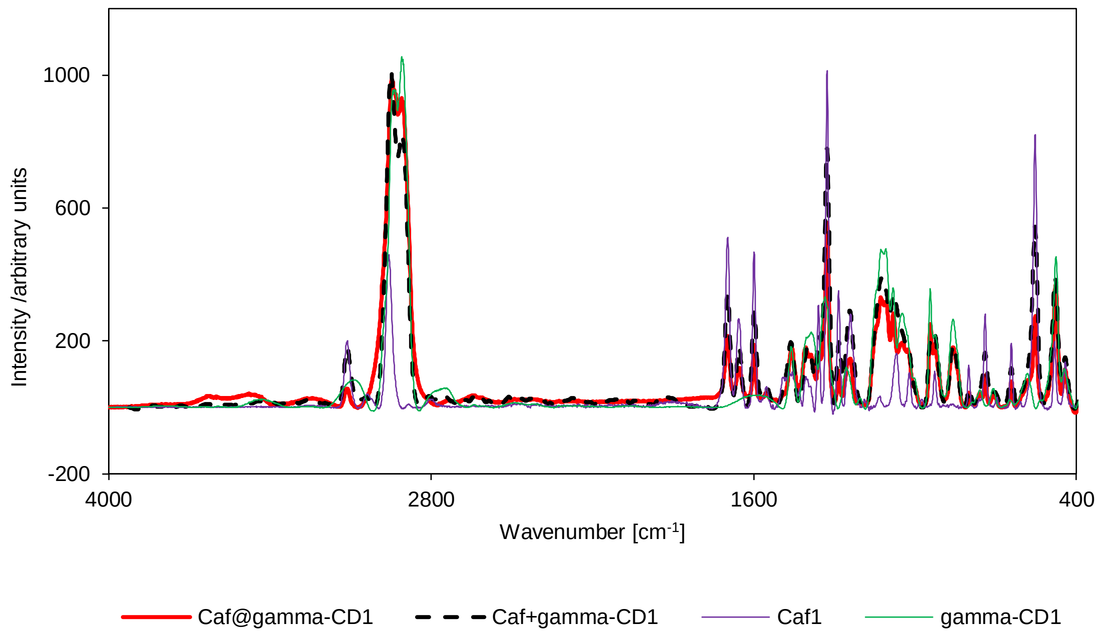

Figure S29. Raman spectra for Caf@γ-CD1, Caf+γ-CD1, Caf1, and γ-CD1.

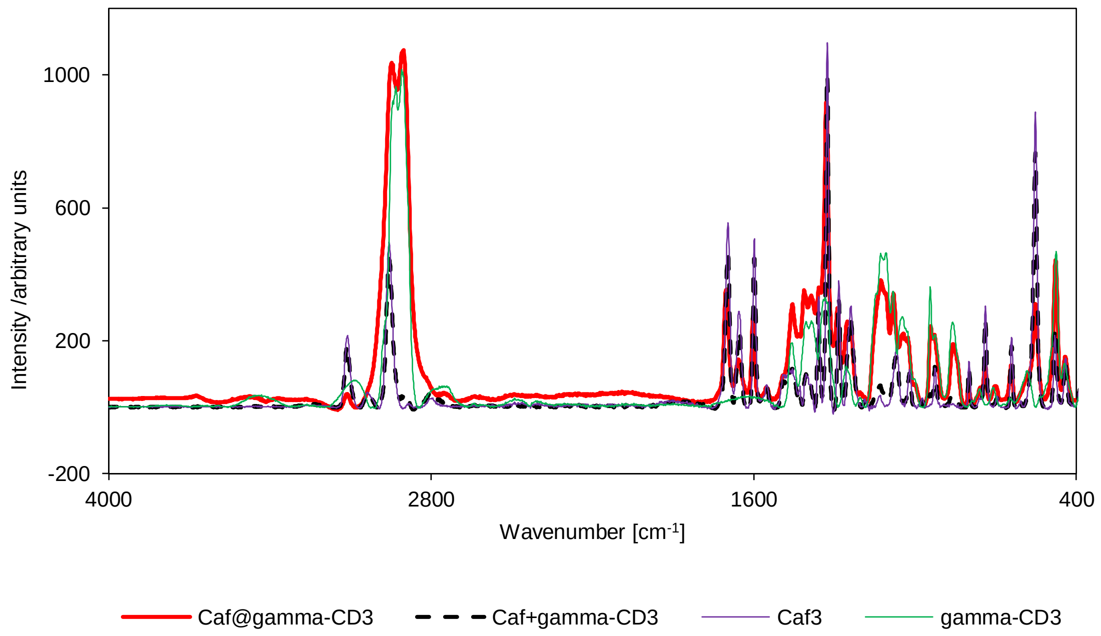

Figure S30. Raman spectra for Caf@γ-CD3, Caf+γ-CD3, Caf3, and γ-CD3.

Results of DSC measurements

Caf1 and Caf3

--- Figures S4, S6, S9, S11, S14, and S16 ---

The DSC thermograms for both Caf1 and Caf3 revealed two endothermic peaks. The first signals at 148°C (31 J·g^{-1}) and 135°C (24.5 J·g^{-1}) for Caf1 and Caf3, respectively, are relatively broad and flat and are probably connected with dehydration. The subsequent peaks are very sharp and are associated with the melting process since the temperatures of 233°C (195 J·g^{-1}) and 230°C (193 J·g^{-1}) for Caf1 and Caf3, respectively, are close to the reported melting points of caffeine [1-3].

α-CD1

--- Figure S4 ---

Three endothermic peaks were collected: at 66°C (16 J·g^{-1}), 157°C (8 J·g^{-1}), and 180°C (270 J·g^{-1}). The first one is broad and flat, the second one is small with a sharp beginning and a poorly formed ending. The second process looks like a jump on the baseline. The third peak is sharp with a distinct beginning and an irregular ending. This could be due to several different processes, *e.g.* melting of a few inequivalent polymorphic forms.

α-CD3

--- Figure S6 ---

This thermogram showed two distinct endothermic peaks: at 78°C (52 J·g^{-1}) and 174°C (256 J·g^{-1}), and also a third one with the beginning at 138°C and a small heat effect (2.3 J·g^{-1}). The last mentioned peak is broad, which is associated with two endothermal processes with a distinct beginning of the first one and two maxima starting at 174°C.

β-CD1 and β-CD3

--- Figures S9 and S11 ---

For β-CD1 one single peak with a sharp beginning and a little broader end was detected at 162°C (255 J·g^{-1}). In the case of β-CD3, two endothermic peaks were observed at 142°C (7.5 J·g^{-1}) and 175°C (493 J·g^{-1}). The first peak has a small heat effect, whereas the second one is rather broad with a sharp beginning of the process.

γ-CD1 and γ-CD3

--- Figures S14 and S16 ---

For both substances, three endothermic processes were observed. In the case of γ-CD1, the following parameters were detected: 40°C (1.7 J·g^{-1}), 184°C (2.2 J·g^{-1}), and 221°C (80 J·g^{-1}). The second process seems to be a jump on the baseline, that is why it is considered to be connected with transitions without heat effects. For γ-CD3 the first process appeared at 104°C (4.1 J·g^{-1}), whereas the second one ranged from 132 to 162°C (23.5 J·g^{-1}) and came from several different phases. The third peak for γ-CD3 was observed at 174°C (413 J·g^{-1}). For both γ-CD1 and γ-CD3 the last mentioned peaks were the most intensive on the thermograms.

Caf+α-CD1

--- Figures S4 and S5 ---

Three endothermic peaks were registered: the first one at 57°C (1.9 $J{\cdot}g^{-1}$) is rather small, broad and flat; the second one is very broad with a poorly detected beginning (ca. 90°C) including two processes whose second maximum has easily ascribable parameters at 133°C (124 $J{\cdot}g^{-1}$); the third one starts as a small exothermic effect with smooth transition into the endothermic process at 234°C (23 $J{\cdot}g^{-1}$) which is distinctly sharp and narrow.

Caf+α-CD3

--- Figures S6 and S7 ---

There are three big processes and two smaller endothermic ones on the DSC thermogram. The first signal is at 78°C (38 $J{\cdot}g^{-1}$), the second one is very small at 99°C (2.2 $J{\cdot}g^{-1}$), the third one is at 141°C (2.4 $J{\cdot}g^{-1}$). The next one is very broad at 162°C (267 $J{\cdot}g^{-1}$) coming from two endothermic transitions with two distinct maxima in spite of a somewhat big overlapping. The last one is at 229°C (28 $J{\cdot}g^{-1}$).

Caf+β-CD1

--- Figures S9 and S10 ---

Four distinct transitions were detected. The first peak with a poorly ascribable beginning (at ca. 68°C) comes from two phases. The endothermic peak of the second phase starts at ca. 104°C (157 $J{\cdot}g^{-1}$). It is the biggest transition for this substance. Other transitions are represented by small endothermic peaks at 154°C (1.8 $J{\cdot}g^{-1}$), 235°C (4.4 $J{\cdot}g^{-1}$), and 268°C (16 $J{\cdot}g^{-1}$).

Caf+β-CD3

--- Figures S11 and S12 ---

There are three processes represented by the following peaks: one at 141°C (476 $J{\cdot}g^{-1}$), very broad till 190°C with two distinct maxima, and the other two at 233°C (8.6 $J{\cdot}g^{-1}$) and 257°C (7 $J{\cdot}g^{-1}$).

Caf+γ-CD1

--- Figures S14 and S15 ---

The thermogram revealed two evident endothermic transformations at 150°C (5 $J{\cdot}g^{-1}$) and 161°C (89 $J{\cdot}g^{-1}$).

Caf+γ-CD3

--- Figures S16 and S17 ---

Four endothermic peaks were observed at 96°C (9.4 $J{\cdot}g^{-1}$), 119°C (7 $J{\cdot}g^{-1}$), 148°C (11 $J{\cdot}g^{-1}$), and 172°C (290 $J{\cdot}g^{-1}$). The last mentioned peak was the most intensive one.

Caf1, α-CD1, and Caf+α-CD1

--- Figure S4 ---

The thermogram of the mixture showed one endothermic peak at 235°C coming from pure Caf1. However, this peak is significantly small, the value of heat changed from 220 to 23 $J{\cdot}g^{-1}$. Moreover, there is no peak at 180°C coming from α-CD1, but a new peak appeared at 120°C which could come from pure Caf1, however, this peak is significantly less intensive. It might be a peak coming from a new phase whose intensity decreased twice in comparison with the intensity of two phases that could form it. Nevertheless, PXRD patterns did not reveal the formation of new phases in the case of all mixtures under investigation. The PXRD patterns of all mixtures were a simple sum of the peaks collected for appropriate pure ingredients.

Caf3, α-CD3, and Caf+α-CD3

--- Figure S6 ---

There are several peaks (the majority of them at the same temperatures) coming from pure components on the thermogram of the mixture. Only one peak from α-CD3 which starts at 173°C moved to lower temperatures (163°C) in the case of a mixture. The intensity of the peak from Caf3 at 230°C decreased significantly from 193 to 34 $J{\cdot}g^{-1}$. The new peak at 163°C could not come from a new phase because the formation of any new phase was excluded according to PXRD measurements.

Caf1, β-CD1, and Caf+β-CD1

--- Figure S9 ---

Similarly as above, the endothermic peak at 235°C from pure Caf1 was significantly less intensive in the case of a mixture (the value of heat decreased from 195 to 4.4 $J{\cdot}g^{-1}$). Several new transformations from 200 to 290°C of low intensities appeared for the mixture (the most intensive one at 268°C (16 $J{\cdot}g^{-1}$)). Moreover, a new broad endothermic peak starting at ca. 70°C (ending at 150°C) could be observed on the thermogram for the mixture. There are no peaks from pure Caf1 at 148°C and β-CD1 at 16°C. Even though new signals appeared on the thermogram for the mixture, the possibility of formation of a new phase was excluded according to PXRD findings.

Caf3, β-CD3, and Caf+β-CD3

--- Figure S11 ---

There are several peaks (the majority of them at the same temperatures) coming from pure ingredients on the thermogram for the mixture. Only the peak from β-CD3 was shifted toward lower temperatures (at ca. 35°C). Furthermore, the peak at 233°C from pure Caf3 was significantly less intensive (the value of heat changed from 193 to 8.6 $J{\cdot}g^{-1}$). A new small and broad (without a sharp beginning and end) endothermic peak appeared at 257°C (7 $J{\cdot}g^{-1}$).

Caf1, γ-CD1, and Caf+γ-CD1

--- Figure S14 ---

There are no peaks from pure Caf1 and γ-CD1 on the thermogram for the mixture. A new peak at 161°C (90 $J{\cdot}g^{-1}$) was revealed. Just before it, there is a small peak at 150°C (5 $J{\cdot}g^{-1}$) which could come

from pure Caf1. However, in this case too, the formation of a new phase (due to the presence of the new peak at 161°C) must be rejected in view of PXRD results.

Caf3, γ-CD3, and Caf+γ-CD3

--- Figure S16 ---

The thermogram of the mixture did not reveal any new peaks of transformations. There are only peaks from pure ingredients, however, they are less sharp and of small intensities. Only the peak from pure γ-CD3 at 174°C is more noticeable with a significantly lower value of heat which equals 290 $J{\cdot}g^{-1}$. There is no peak connected with the melting of pure Caf3.

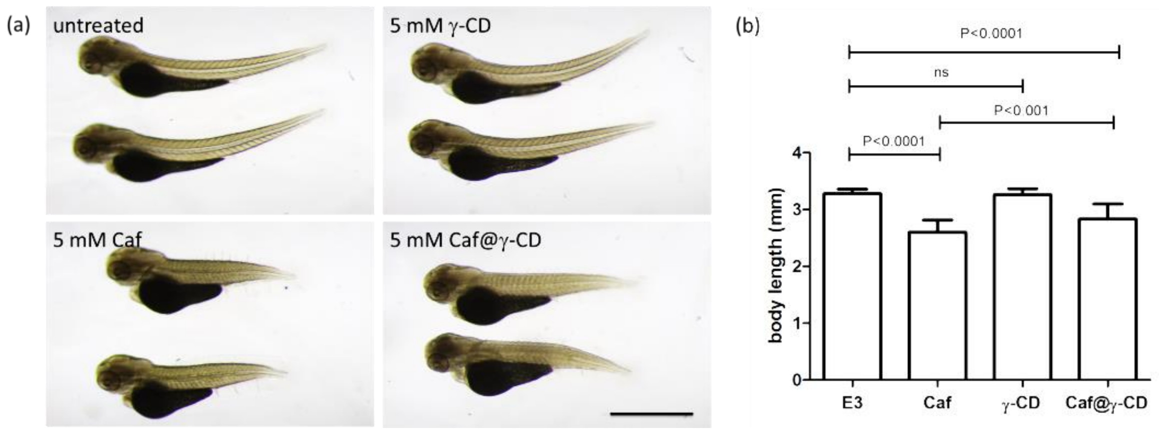

Figure S31. Developmental defects caused by caffeine (Caf). (a) Representative images of PFA-fixed zebrafish embryos. Live embryos were exposed to caffeine, γ-CD or Caf@γ-CD from 36 till 48 hpf. The most severe morphological abnormalities can be noted in embryos exposed to caffeine. Caf@γ-CD had intermediate effect, whereas γ-CD had no effect on embryo development. Lateral view; scale bar, 1 mm; (b) measurements of body length of zebrafish embryos as shown in (a). Statistical significance between two groups was evaluated by the two-tailed unpaired *t*-test using the GraphPad Prism version 5.0 for Windows. Data are presented as mean values ± SD, n > 29 in each group.

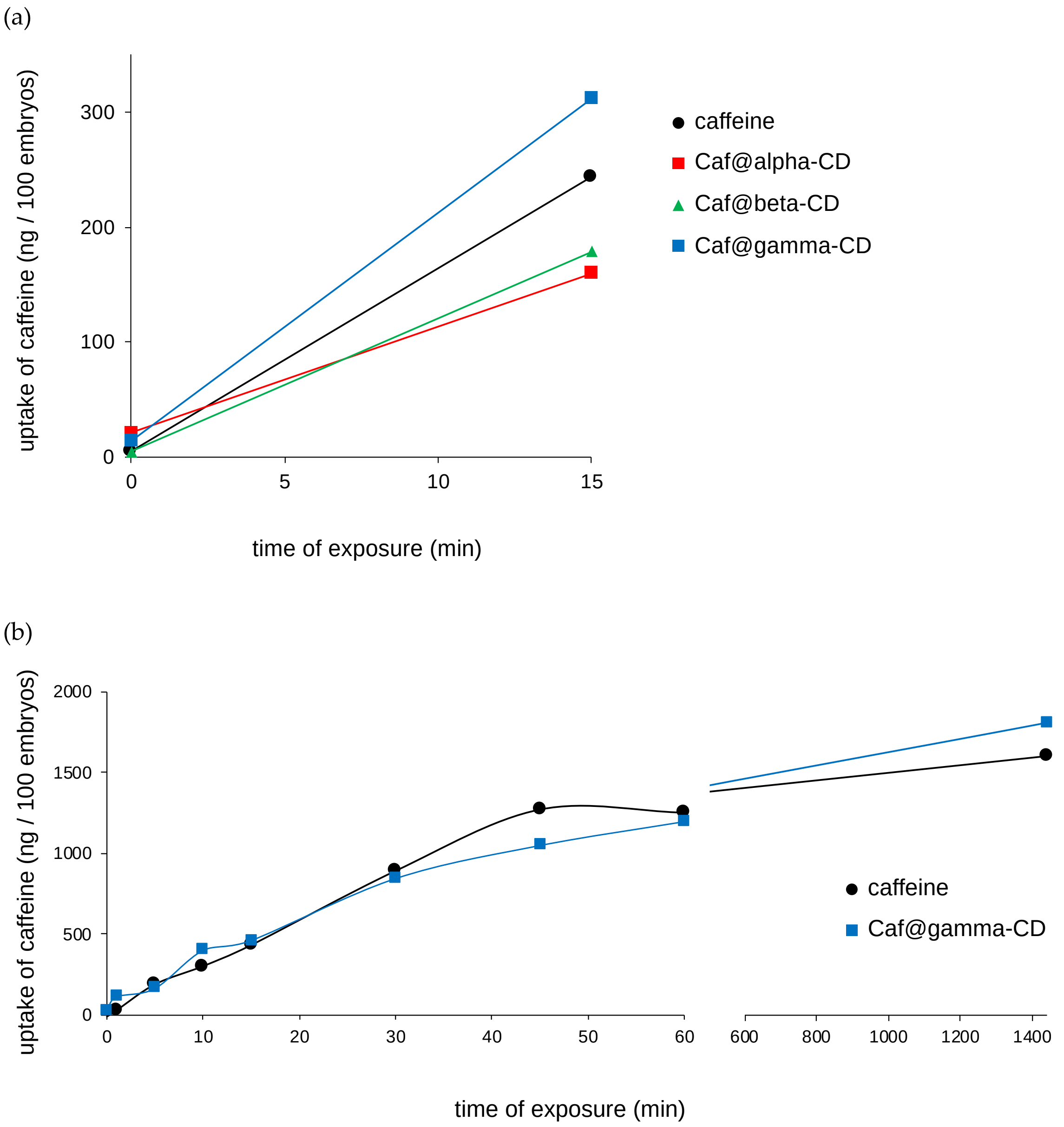

Figure S32. Examples of the caffeine uptake by zebrafish embryos treated from 4 hpf with 50 μM caffeine, Caf@α-CD, Caf@β-CD (a) or Caf@γ-CD (a and b).

References

1. Ferreyra, C.; Ortiz, C.; de Bertorello, M.M. Development and validation of a chromatographic method for the analysis of multicompound pharmaceutical preparations. *Molecules* **2000**, *5*, 574-575, doi:10.3390/50300574.
2. Guo, K.; Sadiq, G.; Seaton, C.; Davey, R.; Yin, Q. Co-Crystallization in the Caffeine/Maleic Acid System: Lessons from Phase Equilibria. *Crystal Growth & Design* **2010**, *10*, 268-273, doi:10.1021/cg900885n.
3. Tanabe, T.; Yamauchi, K.; Kinoshita, M. The N-Alkylation of Xanthine Derivatives with Trialkyl Phosphates. *Bulletin of the Chemical Society of Japan* **1976**, *49*, 3224-3226, doi:10.1246/bcsj.49.3224.
